# Hypoxic glioma-derived exosomal miR-25-3p promotes macrophage M2 polarization by activating the PI3K-AKT-mTOR signaling pathway

**DOI:** 10.1186/s12951-024-02888-5

**Published:** 2024-10-16

**Authors:** Zhiwei Xue, Junzhi Liu, Wenchen Xing, Feiyu Mu, Yanzhao Wu, Jiangli Zhao, Xuchen Liu, Donghai Wang, Jian Wang, Xingang Li, Jiwei Wang, Bin Huang

**Affiliations:** 1 Department of Neurosurgery, Qilu Hospital, Cheeloo College of Medicine and Institute of Brain and Brain-Inspired Science, Shandong University, Jinan, China; 2 Jinan Microecological Biomedicine Shandong Laboratory and Shandong Key Laboratory of Brain Health and Function Remodeling, Jinan, China; 3 Department of Neurosurgery, Qilu Hospital of Shandong University Dezhou Hospital, Dezhou, China; 4 Department of Biomedicine, University of Bergen, Jonas Lies Vei 91, Bergen, 5009 Norway

**Keywords:** Glioma, Exosomes, miRNAs, PHLPP2, AKT-mTOR

## Abstract

**Background:**

Exosomes (EXO) play crucial roles in intercellular communication and glioma microenvironment modulation. Tumor-associated macrophages are more likely to become M2-like type macrophages in the immunosuppressive microenvironment. Here, we aimed to investigate the effects and molecular mechanisms of hypoxic glioma-derived exosomes mediated M2-like macrophage polarization.

**Methods:**

Highly expressed miRNAs in exosomes derived from glioma cells cultured under hypoxia condition compared to normoxic condition were identified through microRNA sequencing. The polarization status of macrophages was determined using qRT-PCR, Western blotting, flow cytometry, and immunohistochemistry. By using RNA-seq, we aimed to identify the downstream target genes regulated by miR-25-3p in macrophages and investigate the mechanistic pathways through which it exerts its effects. The proliferation and migration capabilities of glioma cells were assessed through EdU, Transwell assays, and in vivo experiments.

**Results:**

We found that miR-25-3p was upregulated in the exosomes derived from hypoxic glioma cells and can be transferred to the macrophage. In macrophages, miR-25-3p downregulates the expression of PHLPP2, thereby activating the PI3K-AKT-mTOR signaling pathway, ultimately leading to macrophage M2 polarization. As part of a feedback loop, M2-polarized macrophages can, in turn, promote malignant glioma progression.

**Conclusion:**

Our study reveals that miR-25-3p from hypoxic glioma cells is delivered to macrophages via exosomes as a mediator, promoting M2 polarization of macrophages through the miR-25-3p/PHLPP2/PI3K-AKT signaling pathway. This study suggests that targeted interventions to modulate miR-25-3p expression, transmission, or inhibition of PI3K-AKT pathway activation can disrupt the immune-suppressive microenvironment, providing a novel approach for immunotherapy in gliomas.

**Graphical Abstract:**

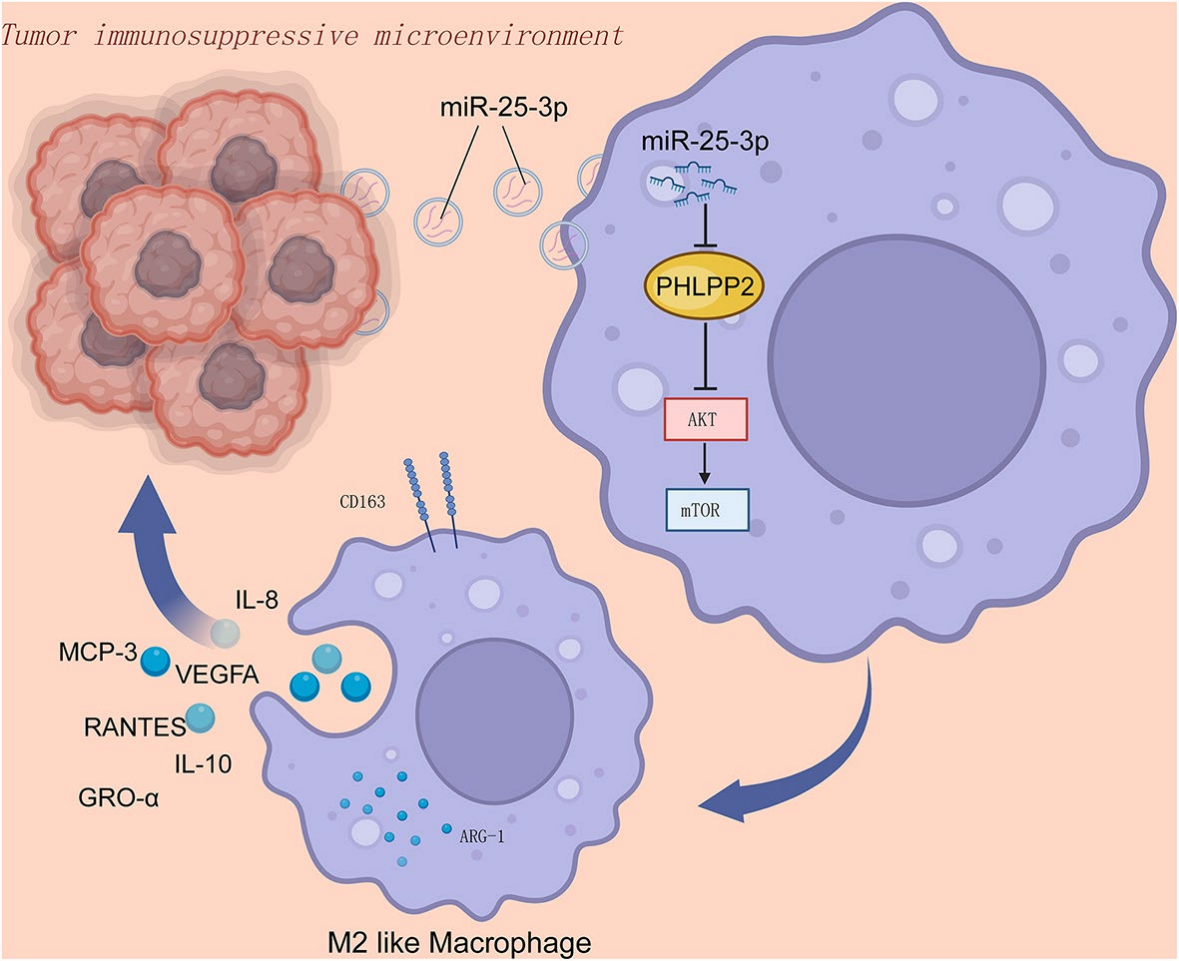

**Supplementary Information:**

The online version contains supplementary material available at 10.1186/s12951-024-02888-5.

## Introduction

Glioblastoma (GBM) is the most prevalent and malignant primary intracranial brain tumor in adults, with a very poor prognosis [1]. Despite surgery and temozolomide chemoradiotherapy, the median survival of GBM patients is still less than 2 years [2, 3]. The ecosystem surrounding GBM is intricate and highly stratified, comprising both tumor and non-tumor components. The tumor microenvironment is made up of immune cells, astrocytes, endothelial cells, and macrophages [4]. Like other solid tumors, GBM also has extensive areas of hypoxia and necrosis. Furthermore, within the glioma microenvironment, macrophages, the predominant immune cells, exhibit a predilection for preferential accumulation in regions characterized by hypoxia, allowing for their subsequent differentiation into distinct cellular subtypes [4, 5]. Interactions between immune cells and tumor cells constitute a critical element in the advancement of tumorigenesis. Specifically, infiltrating macrophages are instrumental in fostering tumor angiogenesis, expansion, and resistance to therapeutic agents through the secretion of an array of cytokines and chemokines [6, 7].

Microglia are the primary cells with immune function in the central nervous system (CNS) and infiltrate in glioblastoma regions [8]. It is reported that microglia was a type of macrophage that is derived from bone marrow, arborized and present in the parenchyma of the central nervous system. Macrophage or microglia can alter their phenotypes and functions in the tumor immune microenvironment [9]. There are two identified types of polarized phenotypes: classically activated macrophages (M1-like type) and alternatively activated macrophages (M2-like type) [10]. It has been confirmed that M2-like type macrophage or microglia significantly promote tumor progression [11–14].

Exosomes (EXO) are a subset of extracellular vesicles (EVs) with a size range of 40 to 160 nm (average 100 nm) in diameter [15]. EXO play vital roles in regulating intercellular communication by delivering their constituents such as DNA, RNAs, proteins and lipids [16–18]. Therefore, EXO contribute to the influence of neoplasia, tumor growth, metastasis and immunomodulation [15, 19]. The hypoxic microenvironment may stimulate tumor cells to generate exosomes and many of these effects are mediated by EV-encapsulated microRNAs (miRNAs) [20–22]. Interestingly, recent studies have reported that therapeutic drugs can be incorporated into isolated exosomes and then delivered to targeted tissues and cells, enabling precise drug delivery [23, 24]. The low toxicity, low immunogenicity, and high engineerability of exosome-mediated drug delivery hold a promising therapeutic future.

MicroRNAs (miRNAs, miR) are ~ 22-nuceotide RNAs that are involved in gene silencing through direct posttranscriptional suppression of mRNA targets or the expressed proteins [25]. The seed sequences of miRNAs are able to complement multiple conserved sites within the 3’ UTR of mRNA, and the mRNAs of most human genes are conserved targets of at least one miRNA [26]. MicroRNA expression profiling showed that microRNA is associated with tumorigenesis and progression [27]. Moreover, numbers of studies have demonstrated that microRNAs can function as potential oncogenes or tumor suppressor genes, with the aim to improve tumor progression and increase cure rates [28–30]. In recent studies, several microRNAs (miRNAs) derived from tumor cells have been implicated in promoting M2 macrophage polarization, a process that contributes to tumor progression and immune evasion. For instance, colorectal cancer (CRC) cell-derived exosomal miR-934 induces M2 polarization by downregulating PTEN expression and activating the PI3K/AKT signaling pathway [31]. Similarly, CRC-derived miR-21-5p and miR-200a synergistically promote M2-like macrophage polarization and PD-L1 expression through the regulation of the PTEN/AKT and SCOS1/STAT1 pathways, leading to decreased CD8 + T cell activity and enhanced tumor growth [32]. In gastric cancer, exosome-derived circATP8A1 induces M2 polarization via the circATP8A1/miR-1-3p/STAT6 axis, thereby driving tumor progression [33]. Hypoxic conditions further exacerbate this phenomenon, as seen in pancreatic cancer, where hypoxic pancreatic cancer cell-derived exosomal miR-301a-3p facilitates M2 polarization through the activation of the PTEN/PI3Kγ signaling pathway [34]. Additionally, miR-25-3p has been shown to promote M2 polarization of macrophages in the context of CXCL12/CXCR4-induced liver metastasis of colorectal cancer and the epithelial-mesenchymal transition and migration of endometrial epithelial cells [35]. microRNA-25-3p encapsulated in ectopic endometrial cells extracellular vesicles can induce the polarization of macrophages towards M2 direction, and the polarized macrophages promote the transformation of epithelial cells into the mesenchyma [36]. These findings highlight the critical role of tumor-derived miRNAs in modulating the tumor microenvironment, particularly through the induction of M2 macrophage polarization, which in turn supports tumor progression and metastasis.

Importantly, recent studies shown that miR-25-3p is implicated in the progression of glioma through its oncogenic role. miR-25 was significantly up-regulated in astrocytoma tissues and glioblastoma cell lines [37–41]. MiR-25-3p promotes glioma cell proliferation and invasion by targeting genes such as NEFL [37], CDKN1C [38], PTEN [41], FBXW7 [42]. Furthermore, miR-25-3p promotes the temozolomide resistance of glioblastoma cells [42]. Additionally, miR-25-3p may influence the tumor microenvironment by modulating interactions between tumor cells and immune cells through impair cGAS-STING activity [43], further driving tumor growth and metastasis.

In this study, we revealed that miR-25-3p, which were delivered by hypoxic glioma exosomes, induced M2-like microglia polarization via activation of the PI3K-AKT pathway, forming a positive feedback loop to promote glioma progression. A deep comprehensive understanding of how glioblastoma cells and microglia develop through exosomes within the tumor microenvironment (TME) could reveal promising new targets for therapeutic intervention.

## Materials and methods

### Patients and samples

Glioma specimens and adjacent normal tissues were obtained from Qilu Hospital of Shandong University (Jinan, China). Samples of human glioma tissues were collected from patients during surgery at Qilu Hospital. Nonneoplastic brain tissue samples were harvested during surgeries performed on patients with traumatic brain injury. Glioma specimens were assessed and diagnosed by two experienced clinical pathologists according to the latest WHO tumor classification. Clinical data of glioma patients involved in our study were presented in Supplementary Table S1.

### Cell lines and cell culture

The T98, U251 and GL261 cell lines were purchased from the American Type Culture Collection (ATCC), and cultured in Dulbecco’s modified Eagle medium (Thermo Fisher Scientific; Waltham, MA, USA) supplemented with 10% fetal bovine serum (FBS; Thermo Fisher Scientific). Patient-derived GBM stem-like cells (GSCs) P3 were previously isolated and characterized from GBM surgical specimens. STR profiles of GBM cells were presented in Supplementary Table S2. GBM#P3 were cultured in Neurobasal medium (Gibco/Thermo Fisher Scientific) containing 2% B-27 Neuro Mix (Thermo Fisher Scientific), 20 ng/mL epidermal growth factor (EGF; PeproTech; East Windsor, NJ, USA), and 10 ng/mL basic fibroblast growth factor (bFGF; PeproTech). The human microglial cell line (HMC3) was obtained from the ATCC. HMC3 cells were cultured in Eagle’s Minimum Essential Medium (EMEM, ATCC30-2003) supplemented with 10% fetal bovine serum (FBS). For exosomes coculture, 1 µg/ml of exosomes was introduced into the recipient cell culture medium, following established protocols [44, 45]. To assess the involvement of macrophages in glioma progression, an in vitro coculture model was employed. Macrophages were cultivated in 6-well plates and subjected to exosomes treatment or transfection. Subsequently, after a 48-hour incubation, glioma cells were seeded in the upper chamber.

### Isolation of PBMCs-derived macrophage

Peripheral blood mononuclear cells (PBMCs) were isolated from healthy volunteers using lymphocyte separation medium and CD14 magnetic beads. Following isolation, the PBMCs were cultured in 1640 medium with M-CSF (50ng/ml) to induce differentiation into macrophages.

### Transfection for siRNA, miRNA mimic, and miRNA inhibitor

For siRNA, miRNA mimics, and miRNA inhibitor transfection, cells were seeded one day before transfection. The transfection was performed when they were at about 70% confluency using Lipofectamine 3000 (Invitrogen) according to the manufacturer’s instructions. The siRNA, miRNA mimics, and miRNA inhibitor were purchased from Genepharma. The sequence of siRNA, miRNA mimics, and miRNA inhibitor were listed in Supplementary Tables S3, S4.

### Isolation and purification of EVs

As for the isolation of normoxic and hypoxic glioma-derived exosomes, GBM cell lines were cultured in DMEM supplemented with 10% exosome-depleted FBS under normoxic (21% O_2_) or hypoxic (1% O_2_) conditions. 50 mL supernatants from cell culture were collected and centrifuged at 500 g for 10 min followed 5,000 g, 10 min, followed by centrifugation at 12,000 g for 20 min to remove large microvesicles. Then, the obtained supernatant was filtered through a 0.22 μm filter followed by ultracentrifugation at 100,000 × g for 70 min in a 70Ti rotor (Beckman, California, USA) [46]. The supernatant was discarded, and pellets were resuspended with 1 ml PBS and frozen at 80 °C for further use. All the centrifugations were conducted at 4 °C.

### Transmission Electron Microscopy (TEM)

Isolated exosomes were loaded onto a carbon-coated electron microscopy grid and examined using transmission electron microscopy (TEM). One drop of glutaraldehyde (3%) was placed on the grids and incubated for 5 min. Then, the grids were washed with three-fold-distilled water for 2 min for a total of ten washes. Next, the grids were processed with uranyl-acetate solution (4%) for 10 min and methylcellulose solution (1%) for 5 min to compare the exosome samples. Grids were dried and observed using a TEM at 80 kV (Hitachi High-Tech Corporation, Tokyo, Japan).

### NTA

The number and size of the exosomes were directly tracked by the Multiple–Laser Zeta View^®^ f-NTA Nanoparticle Tracking Analyzer (NTA) (Particle Metrix, Germany) [47–49]. The samples were diluted 150–3000 times with PBS, which does not contain any nanoparticles, to attain a concentration of 1–20 × 10^8^ particles per milliliter for analysis. Each sample was measured in triplicate at the camera that recorded and tracked each visible particle.

### In vitro uptake of exosomes into cells

Following the manufacturer’s instruction, exosomes were stained with PKH67 Green Fluorescent Cell Linker Kit (Sigma Aldrich, St Louis, USA, Cat#MINI67) and then terminated by adding 1% bovine serum albumin (BSA). PKH67-labeled exosomes pellets were collected by ultracentrifugation and resuspended in an FBS-depleted medium. Then, EVs were co-cultured with HMC3 for 12 h. After washing twice with PBS, cells were fixed with 4% formaldehyde and stained nuclear with DAPI (Beyotime, China), stained cytoskeletons with TRITC Phalloidin (Yeasen, Shanghai, China). The images were obtained using the LeicaSP8 confocal microscope (Leica Microsystems, Wetzlar, Germany). For co-culture experiments, all of our co-culture experiments were conducted under normoxic conditions. exosome concentration was set to 1 µg/ml with a particle concentration of 5 × 10^9^/ml. In vivo experiments, each mouse received 5 × 10^9^ particles in 100 µl per administration.

### qRT-PCR

Total RNA was extracted using the RNA-Quick Purification Kit (Yishan Biotechnology; Shanghai, China) with DNase treatment and reverse-transcription was performed with the ReverTra Ace qPCR RT Kit (Toyobo; Osaka, Japan). cDNA was amplified using SYBR Green Master Mix on the Roche 480II Real Time PCR Detection System (Roche; Basel, Switzerland). The relative expression levels of mRNA were normalized to GAPDH. The primer sequences used are shown in Supplementary Table S5.

### Immunohistochemistry (IHC)

The animals were intracardially perfused using PBS and 4% paraformaldehyde (PFA). The brains were removed and used to make paraffin sections. Immunohistochemistry was performed following the instructions provided with the biotin-streptavidin-peroxidase and diaminobenzidine kits (SP9000, Zhongshan Golden Bridge Corp., Beijing, China). The slides were scanned by WISLEAP (WS-10) and visualized by NDP view (Version 2.8.24). Analyses of tissue immunohistochemistry were done using ImageJ/FIJI software (Version 1.53c). Staining was evaluated independently to determine the histological score according to the proportion of positive staining cells and staining intensity. The primary antibodies used are shown in Supplementary Table S6.

### Western blot

Total proteins were extracted with RIPA lysis buffer containing a cocktail of protease inhibitors and phosphatase inhibitors (Beyotime, China). The concentration of proteins was determined with the Enhanced BCA Protein Assay Kit (Beyotime) according to the manufacturer’s instructions. Equal amounts of proteins per sample (20 µg) were separated by SDS‒PAGE and then electrotransferred onto PVDF membranes (Merck Millipore; Billerica, MA, USA). Membranes were blocked for 10 min in QuickBlock^™^ Blocking Buffer (Beyotime), incubated with primary antibodies overnight at 4 °C, and followed by incubation with an HRP-conjugated secondary antibody (ZSGB-BIO; Beijing, China) for 1 h. ECL (Merck Millipore) was utilized to detect immunoreactivity, and GAPDH served as a loading control. The primary antibodies used are listed in Supplementary Table S6.

### Transwell assays

For transwell assays, treated HMC3 cells (3 × 10^4^ cells/500 µL) according to the labeled in the article, were seeded and allowed to adhere in 24-well plates. Subsequently, GBM#P3 cells (3 × 10^4^ cells/300 µL) were placed in the upper chambers. Chambers were incubated at 37 °C for 24-48 h, and the cells migrating through the membranes were fixed with 4% paraformaldehyde and stained with crystal violet. Brightfield microscopy images were captured, and the cells were counted by Image J (Version 1.53c).

### Flow cytometry

To detect CD11b^+^CD163^+^ macrophages, anti-CD11b-APC (Abcam, USA), anti-CD163-PE (Abcam, USA) and anti-CD206-FITC (Proteintech, China) were used to stain cells. Isotype controls were run in parallel. Flow cytometry was performed using the Agilent NovoCyte flow cytometer.

### Cytokine array

Cytokine array analysis was performed on supernatants from EXO-P3N or EXO-P3H-treated HMC3 cells with the Human Cytokine Antibody Array (Abcam, Cambridge, MA, USA) according to the manufacturer’s instructions. The grey value of each target was calculated with ImageJ (ImageJ 1.53c, National Institutes of Health; Bethesda, MD, USA) and normalized to the positive control.

### ELISA

Human elisa kit of VEGF, TNF-α, RANTES, MCP-3, IL-10, IL-8 and GRO-α were obtained from proteintech. ELISA assays were performed according manufacturer’s instructions.

### Dual-luciferase reporter assay

The wildtype and mutant 3’UTR region of PHLPP2 were cloned into the vector PmirGLO [luc]. Indicated plasmids and miR-25-3p were co-transfected to the HEK293T using the Lipofectamine 2000 according to the standard Lipofectamine 2000 protocol (Invitrogen). After 48 h, the cells were collected, and the activities of Renilla and firefly luciferases were assessed on a dual-luciferase assay luminometer (Promega; Fitchburg, WI, USA). The activity of the Renilla luciferase gene was used to normalize the firefly luciferase activity. Each experiment was carried out in triplicate, and statistical analysis was performed on the luminescence values of each group.

### EdU Assay

For the EdU assay, cells were treated according to the standard protocols of the Yefluor 488 EdU Imaging Kits (Yeasen Biotechnology). Representative images were captured with a Leica inverted fluorescence microscope. The ratio of EdU-positive cells (red or green) to Hoechst-positive cells (blue) was used to calculate the cell proliferation rate.

### miRNA-seq

Briefly, total RNA was extracted using Trizol reagent (Invitrogen, CA, USA) following the manufacturer’s procedure. The total RNA quantity and purity were analysis of Bioanalyzer 2100 (Agilent, CA, USA) with RIN number > 7.0. Approximately 1 ug of total RNA were used to prepare small RNA library according to protocol of TruSeq Small RNA Sample Prep Kits (Illumina, San Diego, USA). And then we performed the single-end sequencing (36–50 bp) on an Illumina Hiseq2500 at the LC-BIO (Hangzhou, China) following the vendor’s recommended protocol. miRNA differential expression based on normalized deep-sequencing counts was analyzed by selectively using Fisher exact test, Chi-squared 2 × 2 test, Chi-squared nXn test, Student t test, or ANOVA based on the experiments design. The significance threshold was set to be 0.01 and 0.05 in each test.

### RNA-seq

HMC3 mRNA sequencing was performed by LC Bio Corporation (Hangzhou, China). Bioinformatics analysis was performed with the OmicStudio tools at https://www.omicstudio.cn/tool. Differential gene expression was identified on the basis of a fold change (FC; |log2(FC)|) of > 1 and a *P*<0.05.

### Animal studies

To eliminate experimental error as much as possible, BALB/c nude mice (male, 4 weeks; GemPharmatech; Nanjing, China) were randomly divided into groups of 5 mice each. GBM#P3 cells expressing luciferase (5 × 10^5^ cells suspended in 10 µL PBS) and different treated HMC3 cells (1 × 10^5^ cells suspended in above 10 µL tumor cell suspension) were transplanted into the frontal lobes of the nude mice with a stereotactic tool (KDS310, KD Scientific; Holliston, MA, USA). Tumor-bearing mice were intravenously injected of PBS, EXO-P3N, or EXO-P3H in the three groups of mice was performed every two days. GL261 cells expressing luciferase (5 × 10^5^ cells suspended in 10 µL PBS) were transplanted into the frontal lobes of the C57 mice with a stereotactic tool (KDS310, KD Scientific; Holliston, MA, USA). Tumor-bearing mice were intravenously injected of PBS, EXO-GL261N, or EXO-GL261H in the three groups of mice was performed every two days. Each mouse received 5 × 10^9^ particles in 100 µl per administration. Tumor growth was examined once each week with bioluminescence imaging (IVIS Spectral In Vivo Imaging System, PerkinElmer; Hopkinton, MA, USA). The animals were euthanized with anesthetics when they showed any signs of persistent discomfort, such as severe hunched posture, reduced movement, apathy, leg dragging, or weight loss of greater than 20%. The mouse brains were perfused, and the brain tissues were sectioned for HE/IHC analysis. The mice that were used to slice the brain tissue were killed at 21 days. In short, the mice were anesthetized and placed on the operating table. PBS and 4% paraformaldehyde were used for perfusion through the left ventricle, and the fluid flowed out of the right auricle. Then the brains of the mice were fixed in 4% paraformaldehyde, and tissue sections were performed after paraffin embedding.

## Results

### Isolation and characterization of glioma cells derived exosomes

**Fig. 1:**
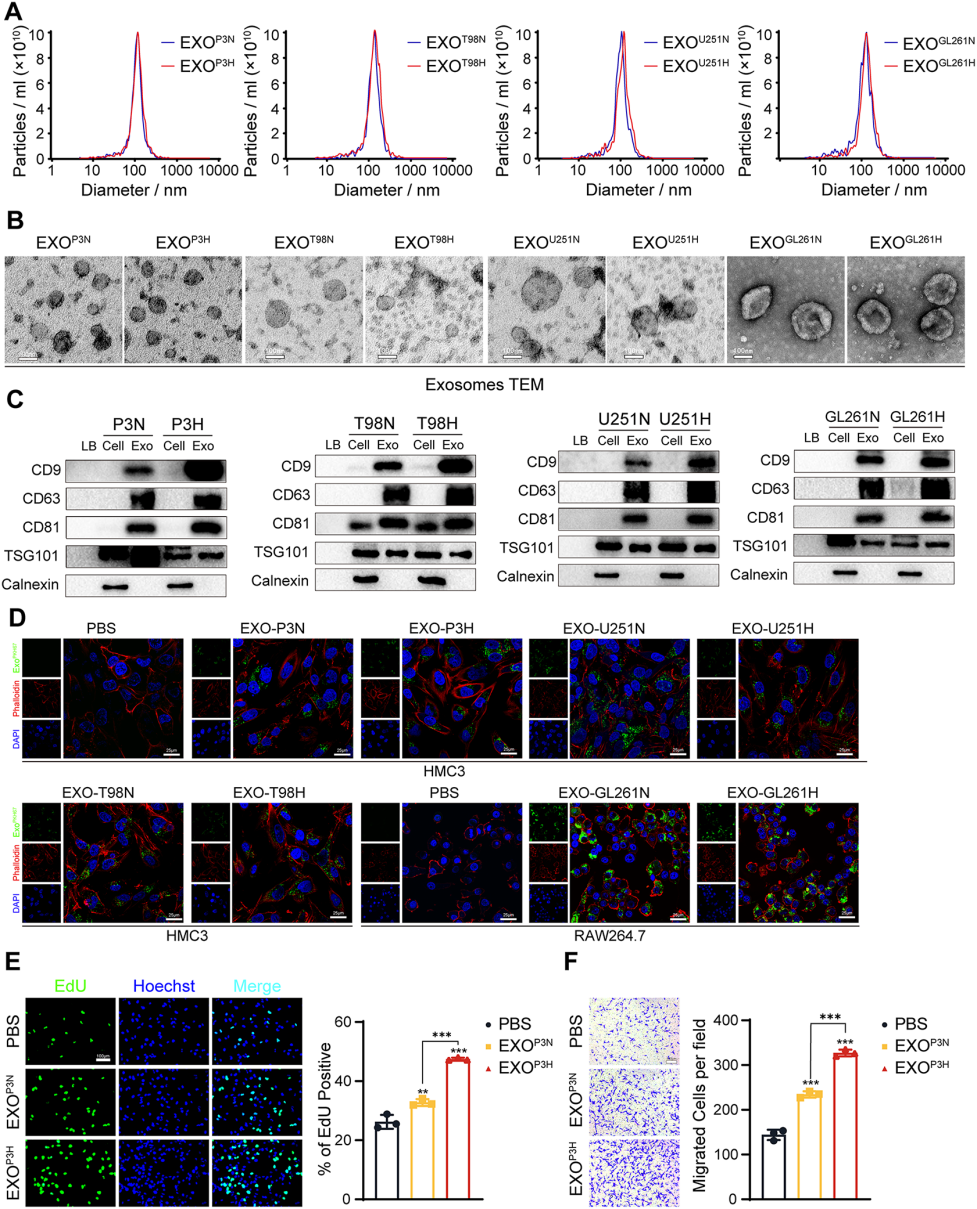
Isolation and characterization of glioma cells derived exosomes. (**A**) Nanoparticle Tracking Analysis (NTA) of normoxic and hypoxic GBM#P3, T98, U251 and GL261-derived exosomes, which purified by serial ultracentrifugation from cell culture supernatants. (**B**) Transmission Electron Microscope (TEM) of normoxic and hypoxic GBM#P3, T98, U251 and GL261-derived exosomes. Scale bar = 100 nm. (**C**) Western blotting analysis for exosomal markers CD9, CD63, CD81, TSG101 and endoplasmic reticulum marker Calnexin of normoxic and hypoxic GBM#P3, T98, U251 and GL261-derived exosomes Loading Buffer (LB) serve as negative control. (**D**) Exosome uptake experiments were visualized by confocal microscopy, which stained the nucleus with DAPI and the cytoskeleton with TRITC-Phalloidin, proved normoxic and hypoxic GBM#P3, T98, U251 and GL261-derived exosomes were extracted, added to cell culture medium and uptaken by HMC3 and RAW264.7 cells. Scale bar = 25 μmy. (**E**) EdU assay was used to evaluate the proliferation of GBM#P3 co-cultured with HMC3 treated by PBS, EXO^P3N^ or EXO^P3H^. The results were quantified. Scale bar = 100 μm. (**F**) The migratory capacity of GBM#P3 co-cultured with HMC3 treated by PBS, EXO^P3N^ or EXO^P3H^. The results were quantified. Scale bar = 100 μm. Data are shown as mean ± SD. *P < 0.05, **P < 0.01, ***P < 0.001

We isolated exosomes from the supernatants of GBM cell lines T98, U251, GBM primary cell GBM#P3 and GL261 under both normoxic and hypoxic conditions. The Nanosight Tracking Analysis (NTA) results revealed that the exosomes primarily exhibited a diameter distribution around 100 nm (Fig. 1A). Transmission electron microscopy (TEM) revealed that the exosomes we isolated exhibited a typical cup-shaped morphology with diameters ranging from 40 to 160 nm (Fig. 1B). Western blot analysis of exosome surface markers demonstrated the presence of CD9, CD63, CD81, and TSG101, while Calnexin was not detected (Fig. 1C). When coculturing PKH67-labeled exosomes derived from GBM#P3, T98, and U251 with human microglial cells (HMC3) or PKH67-labeled exosomes derived from GL261 with RAW264.7 (Figure S1A), we observed a significant uptake of exosomes by HMC3 or RAW264.7, indicating their cellular internalization (Fig. 1D).

To investigate the effects of glioblastoma-derived exosomes under hypoxic conditions on microglial cell or macrophages and their subsequent impact on glioblastoma cell progression, HMC3 were separately treated with PBS, EXO^P3N^ (Exosomes from normoxic P3 cells), EXO^P3H^ (Exosomes from hypoxic P3 cells) or EXO^T98N^ (Exosomes from normoxic T98 cells), EXO^T98H^ (Exosomes from hypoxic T98 cells) or EXO^U251N^ (Exosomes from normoxic U251 cells), EXO^U251H^ (Exosomes from hypoxic U251 cells) and RAW264.7 were separately treated with PBS, EXO^GL261N^ (Exosomes from normoxic GL261 cells), EXO^GL261H^ (Exosomes from hypoxic GL261 cells) for 24 h, followed by coculturing with GBM#P3, T98, U251 orGL261 cells (Figure S1B, C). After 48 h, changes in the proliferation capacity of GBM#P3 cells were assessed through the EdU assay, while their migration ability was evaluated via the Transwell assay. The results indicated that microglial and macrophages treated with EXO under hypoxic conditions significantly promoted the glioma cells proliferation (Fig. 1E, Figure S1D, F, H) and migration (Fig. 1F, Figure S1E, G, I) of glioma cells. These results suggested that exosomes secreted by GBM under hypoxic conditions can modulate the phenotypic of microglial cells and macrophage, and the affected microglial cells or macrophage, in turn, can facilitate GBM progression.

#### EXO under hypoxic condition promoted microglial and macrophage M2 polarization

**Fig. 2:**
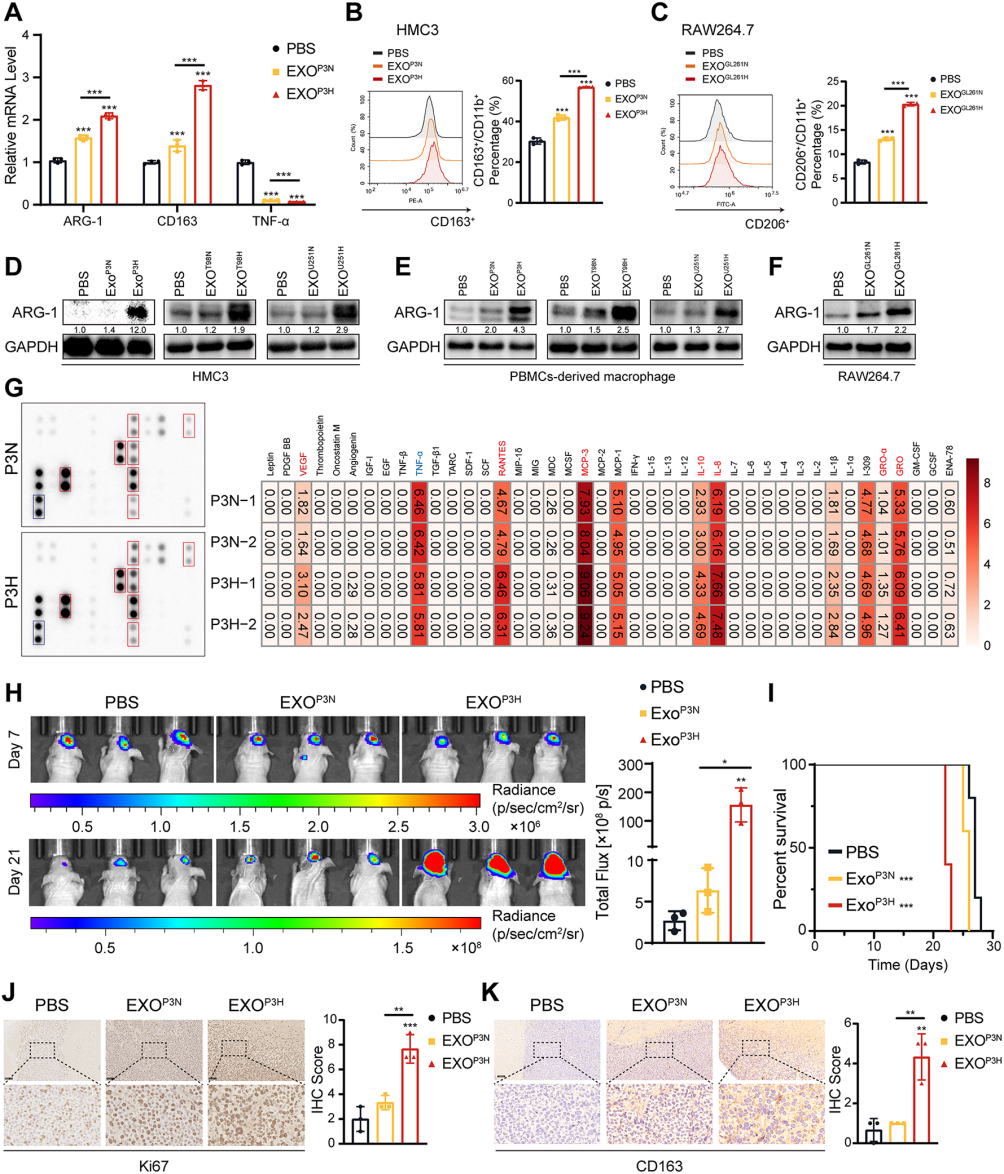
EXO-P3H promote HMC3 M2 polarization. (**A**) qRT-PCR showed the mRNA expression levels of CD163, ARG-1 and TNF-α in HMC3 treated with PBS, EXO-P3N, and EXO-P3H 48 h. Repeat three times for each sample. (**B**) Flow cytometry to detect the proportion of CD163 positive HMC3 cells following treatment with PBS, EXO-P3N, and EXO-P3H 48 h. Repeat three times for each sample. (**C**) Flow cytometry to detect the proportion of CD206 positive RAW264.7 cells following treatment with PBS, EXO-GL261N, and EXO-GL261H 48 h. Repeat three times for each sample. (**D**) Representative Western blot images and quantification of ARG-1 levels in HMC3 treated as indicated. Repeat three times for each sample. (**E**) Representative Western blot images and quantification of ARG-1 levels in PBMCs-derived macrophage treated as indicated. Repeat three times for each sample. (**F**) Representative Western blot images and quantification of ARG-1 levels in RAW264.7 treated as indicated. Repeat three times for each sample. (**G**) Human cytokine antibody array showing expression levels of cytokines detected in supernatants from HMC3 treated with EXO-P3N or EXO-P3H. (**H**) Bioluminescence imaging and quantification of tumors derived from GBM#P3 luciferase expressing cells and HMC3 that differently treated orthotopically implanted into mice. Each mouse received 5 × 10^9^ EV particles in 100 µl per administration. Representative images on day 7 and 21 post-implantation are shown. (**I**) Kaplan-Meier survival curve for mice intravenously injected with PBS, EXO-P3N or EXO-P3H. (**J**) Representative images of IHC for Ki67 expression levels and quantification in sections from the indicated GBM#P3 xenograft. Scale bar = 100 μm. Repeat three times for each sample. (**K**) Representative images of IHC for CD163 expression levels and quantification in sections from the indicated GBM#P3 xenograft. Scale bar = 100 μm. Repeat three times for each sample. Data are shown as mean ± SD. *P < 0.05, **P < 0.01, ***P < 0.001

As widely recognized, M2-like phenotype microglial and macrophages promote tumor progression, while M1-like phenotype can inhibit tumor advancement. Therefore, we subsequently examined the impact of EXO under hypoxic condition on the phenotype of microglial cells and macrophage. The qRT-PCR results revealed that HMC3, Peripheral Blood Mononuclear Cells-derived macrophage (PBMCs-derived macrophage), and RAW264.7 cocultured with EXO under hypoxic condition exhibited a significant upregulation of M2-like phenotype markers, such as ARG-1 and CD163, while the expression of the M1-like phenotype marker TNF-α markedly decreased (Fig. 2A, Figure S2A-F). And consistent with this, flow cytometry results also indicated that EXO-P3H promoted HMC3 cells M2 polarization (Fig. 2B, C; Figure S2G-I). Although EXO from glioma cells under normoxia condition also led to an increase in ARG-1 and CD163, the effect of EXO from glioma cells under hypoxic condition was more pronounced. The expression levels of ARG-1 were further validated by Western blot analysis (Fig. 2D-F), Furthermore, cytokine array experiments suggested that EXO-P3H can stimulate the secretion of pro-tumorigenic factors, including VEGF, RANTES, MCP-3, IL-10, IL-8, GRO-α and GRO, by HMC3 cells, while the TNF-α was downregulated, thereby promoting the malignant progression of GBM (Fig. 2G, Figure S3A). These results were further validated by ELISA (Figure S3B). In order to investigate the in vivo impact of EXO from glioma under hypoxic condition on M2-like microglial polarization, HMC3 cells treated with PBS, EXO-P3N, or EXO-P3H were co-implanted with glioma cells into the brains of nude mice. Subsequently, injections of PBS, EXO^P3N^, or EXO^P3H^ were administered every two days. Tumor sizes were assessed using bioluminescence imaging on the 7- and 21- days post-implantation. It was evident that tumors displayed significant enlargement in the EXO-P3H group (Fig. 2H). Furthermore, animals bearing gliomas co-implanted with EXO-P3H-treated macrophages exhibited a reduced survival rate compared to the other experimental groups, the median survival times for mice in the PBS treatment group, P3N treatment group, and P3H treatment group were 27 days, 26 days, and 22 days (Fig. 2I). Interestingly, IHC staining showed that the mice treated with EXO-P3H exhibited higher expression of Ki-67 and CD163 (Fig. 2J, K). Furthermore, GL261 cells were implanted into the brains of c57 mice. Subsequently, injections of PBS, EXO^GL261N^, or EXO^GL261H^ were administered every two days. The results are consistent with those mentioned above (Figure S4A-D). These results indicated that in vivo, EXO from glioma cells under hypoxic condition can promote M2 polarization of microglial and macrophages, resulting in an immunosuppressive microenvironment within the GBM, thereby facilitating GBM proliferation.

#### Hypoxic GBM derived exosomes leaded to M2 polarization by supplementing miR-25-3p

**Fig. 3:**
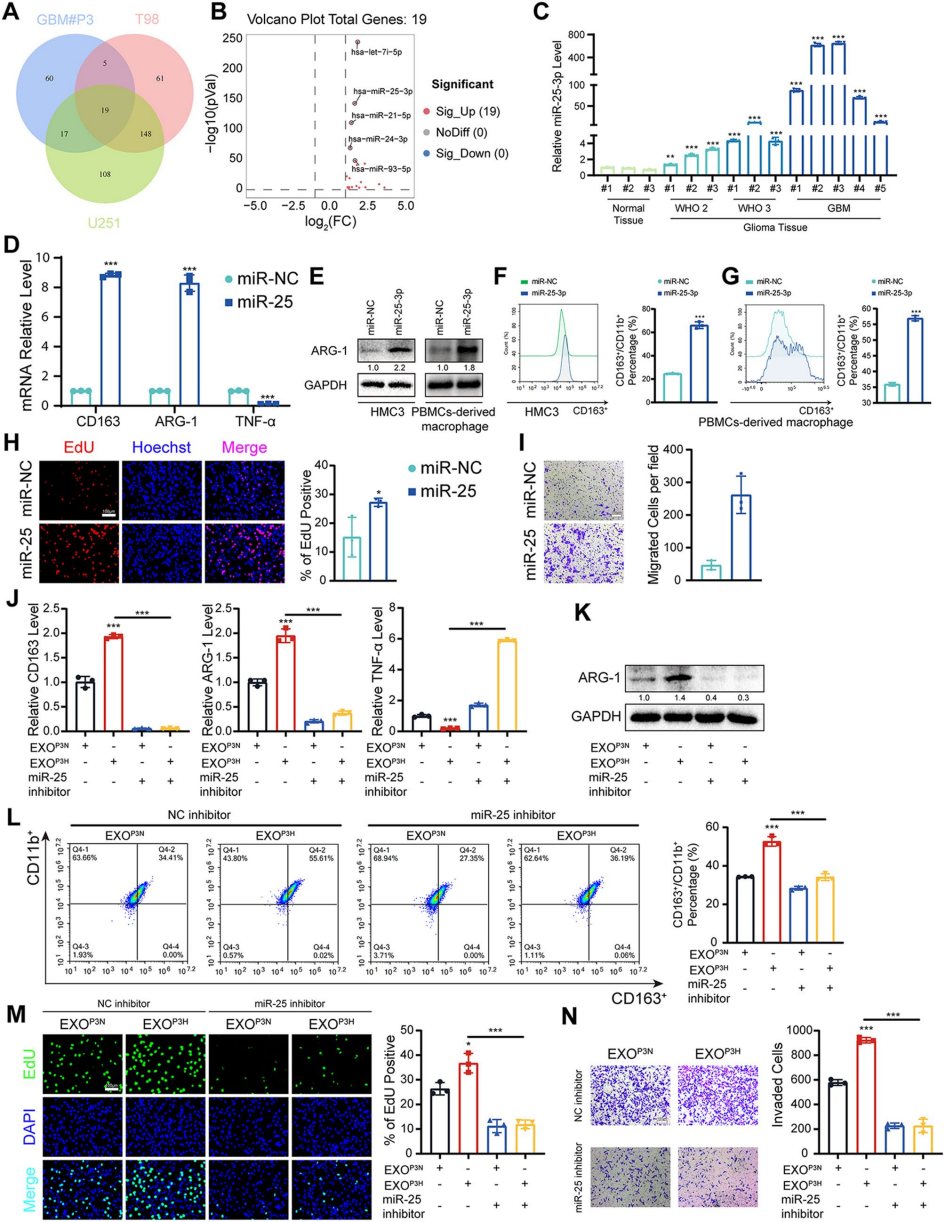
Hypoxic GBM derived exosomes leaded to macrophage M2 polarization by supplementing miR-25-3p. (**A**) Intersecting highly expressed microRNAs in the exosomes of T98, U251, and P3 under hypoxic conditions compared to normoxic conditions. (**B**) The distribution-statistical differences of microRNA obtained by taking intersections in GBM#P3 (normoxic and hypoxic) were analyzed by Volcano plots. (**C**) qRT-PCR showed the expression levels of miR-25-3p in normal tissue and glioma tissues of different WHO grades. Repeat three times for each sample. (**D**) qRT-PCR showed the mRNA expression levels of CD163, ARG-1 and TNF-α in HMC3 transfected with miR-NC or miR-25-3p. Repeat three times for each sample. (**E**) Representative Western blot images and quantification of ARG-1 levels in HMC3 and PBMCs-derived macrophage transfected with miR-NC or miR-25-3p. Repeat three times for each sample. (**F**) Flow cytometry to detect the proportion of CD163 positive HMC3 transfected with miR-NC or miR-25-3p. Repeat three times for each sample. (**G**) Flow cytometry to detect the proportion of CD163 positive PBMCs-derived macrophage transfected with miR-NC or miR-25-3p. Repeat three times for each sample. (**H**) EdU assay was used to evaluate the proliferation of GBM#P3 co-cultured with HMC3 transfected with miR-NC or miR-25-3p. Scale bar = 100 μm. Repeat three times for each sample. (**I**) The migratory capacity of GBM#P3 co-cultured with HMC3 transfected with miR-NC or miR-25-3p. The results were quantified. Scale bar = 100 μm. Repeat three times for each sample. (**J**) qRT-PCR showing the mRNA expression levels of CD163, ARG-1 and TNF-α in HMC3 treated as indicated. The working concentration of miR-25 inhibitor for treating the cells was 0.06 µM. Repeat three times for each sample. (**K**) Representative Western blot images and quantification of ARG-1 levels in HMC3 treated as indicated. The working concentration of miR-25 inhibitor for treating the cells was 0.06 µM. Repeat three times for each sample. (**L**) Flow cytometry to detect the proportion of CD163 positive HMC3 treated as indicated. Repeat three times for each sample. The working concentration of miR-25 inhibitor for treating the cells was 0.06 µM. Repeat three times for each sample. (**M**) EdU assay was used to evaluate the proliferation of GBM#P3 co-cultured with HMC3 treated as indicated. The working concentration of miR-25 inhibitor for treating the cells was 0.06 µM. Scale bar = 100 μm. Repeat three times for each sample. (**N**) The migratory capacity of GBM#P3 co-cultured with HMC3 treated as indicated. The results were quantified. The working concentration of miR-25 inhibitor for treating the cells was 0.06 µM. Scale bar = 100 μm. Repeat three times for each sample. Data are shown as mean ± SD. *P < 0.05, **P < 0.01, ***P < 0.001

To identify which components played a crucial role under hypoxic culture, we conducted separate comparisons of microRNAs under hypoxic conditions compared to normoxic conditions for P3, T98 and U251. High-expression miRNAs obtained by taking intersection (Fig. 3A), and their statistical-distribution differences were presented by volcano plots (Fig. 3B). Previous studies have reported that miR-25-3p can be delivered to endothelial cells through exosomes, promoting angiogenesis and altering the microenvironment to facilitate tumor metastasis [50]. Interestingly, we found that miR-25-3p expression was elevated in hypoxic glioma cells and glioma tissues (Fig. 3C, Figure S5A, B). qRT-PCR showed that the relative expression of miR-25-3p in exosomes was significantly increased under hypoxic conditions (Figure S5C). Moreover, HMC3 cells co-cultured with hypoxic exosomes had significantly higher miR-25-3p levels than the PBS and normoxic groups (Figure S5D-F). These findings indicated that miR-25-3p is highly expressed in EXO from glioma cells under hypoxic condition and can be transferred from cell to cell via exosomes.

Next, we validated whether miR-25-3p could promote M2-like polarization in HMC3 and PBMCs-derived macrophage. After overexpression of miR-25-3p (Figure S5G, H), we extracted total RNA from HMC3 and PBMCs-derived macrophage and performed qRT-PCR. The expression of ARG-1 and CD163 were significantly increased, while the expression of TNF-α was markedly decreased (Fig. 3D, Figure S5I). Next, ARG-1 expression and CD163 expression were investigated by western blotting (Fig. 3E) and flow cytometry (Fig. 3F, G). EdU and Transwell assays showed that co-culture with HMC3 cells overexpressing miR-25-3p significantly enhanced the proliferation and migration of GBM#P3 cells (Fig. 3H, I). However, when we treated HMC3 with miR-25-3p inhibitor (0.06 µM) (Figure S5J), the M2 polarization of HMC3 was reversed confirmed by qRT-PCR, western blot, and flow cytometry analyses (Fig. 3J-L). Consistently, the proliferation and migration capacity were also reduced with addition of miR-25-3p inhibitor (Fig. 3M, N). These results suggested that exosomal miR-25-3p derived from hypoxic GBM promoted HMC3 M2 polarization.

#### MiR-25-3p enhances M2 polarization via PI3K-AKT pathways activation in microglial and macrophage

**Fig. 4:**
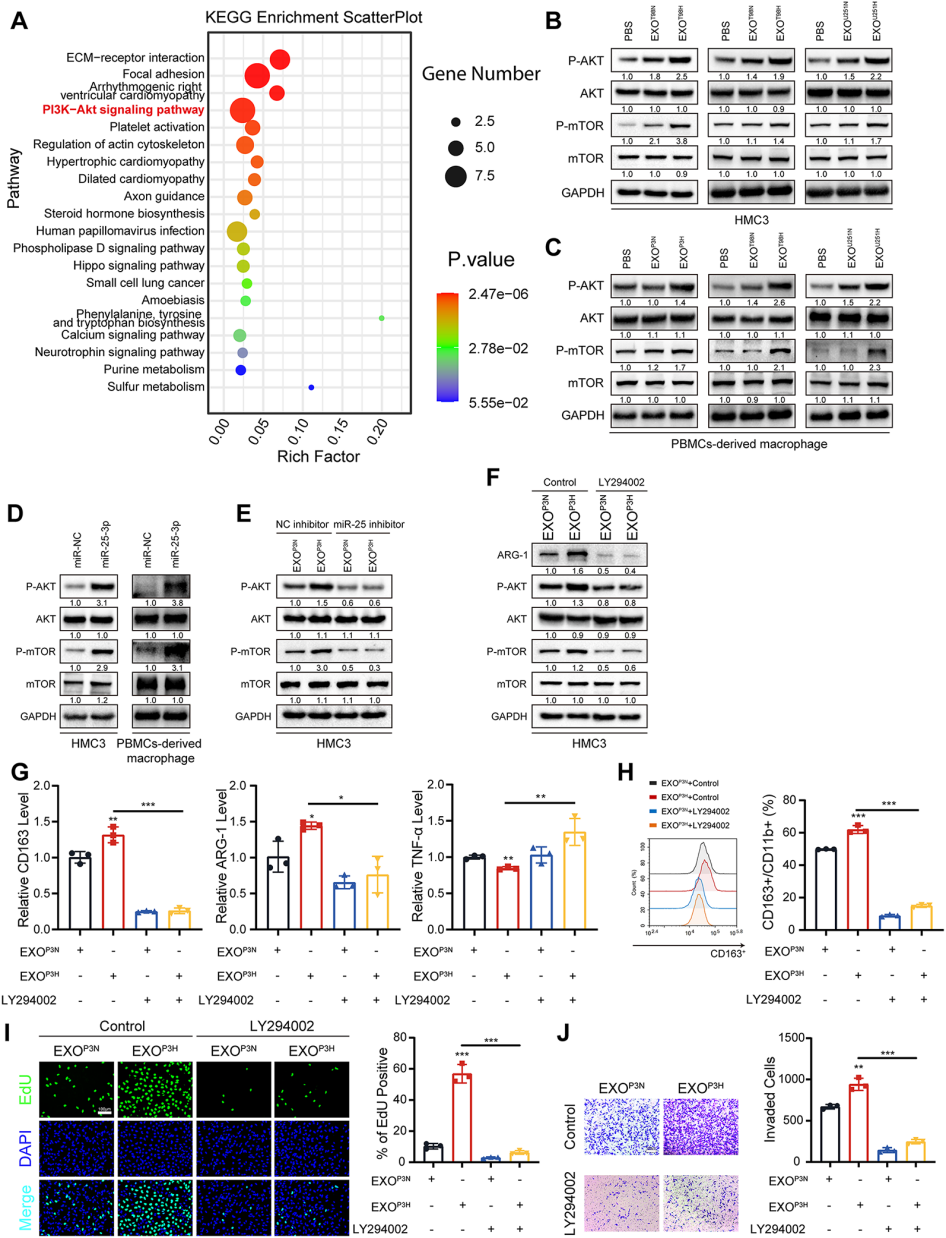
MiR-25-3p enhances M2 polarization via PI3K-AKT pathways activation in macrophages. (**A**) KEGG enrichment was analyzed using the differentially expressed genes that in HMC3 transfected with miR-NC or miR-25-3p and visualized by Scatter Plot. (**B**) Representative Western blot images and quantification of P-AKT, AKT, P-mTOR, mTOR levels in HMC3 treated as indicated. Repeat three times for each sample. (**C**) Representative Western blot images and quantification of P-AKT, AKT, P-mTOR, mTOR levels in PBMCs-derived macrophage treated as indicated. Repeat three times for each sample. (**D**) Representative Western blot images and quantification of P-AKT, AKT, P-mTOR, mTOR levels in HMC3 and PBMCs-derived macrophage treated as indicated. Repeat three times for each sample. (**E**) Representative Western blot images and quantification of P-AKT, AKT, P-mTOR, mTOR levels in HMC3 treated as indicated. The working concentration of miR-25 inhibitor for treating the cells was 0.06 µM. Repeat three times for each sample. (**F**) Representative Western blot images and quantification of ARG-1, P-AKT, AKT, P-mTOR, mTOR levels in HMC3 treated as indicated. Repeat three times for each sample. (**G**) qRT-PCR showed the mRNA expression levels of CD163, ARG-1 and TNF-α in HMC3 treated as indicated. Repeat three times for each sample. (**H**) Flow cytometry to detect the proportion of CD163 positive HMC3 treated as indicated. Repeat three times for each sample. (**I**) EdU assay was used to evaluate the proliferation of GBM#P3 co-cultured with HMC3 treated as indicated. Scale bar = 100 μm. Repeat three times for each sample. (**J**) The migratory capacity of GBM#P3 co-cultured with HMC3 treated as indicated. The results were quantified. Scale bar = 100 μm. Repeat three times for each sample. Data are shown as mean ± SD. *P < 0.05, **P < 0.01, ***P < 0.001

To explore the mechanism through which miR-25-3p induces M2 polarization in microglial and macrophages, we conducted RNA-seq on HMC3 cells overexpressing miR-25-3p and miR-NC. Subsequently, the differentially expressed genes were subjected to KEGG enrichment analysis, revealing a significant enrichment in the PI3K-AKT signaling pathway (Fig. 4A). Previous studies have also suggested that the activation of the PI3K-AKT signaling pathway can lead to macrophage M2 polarization [31, 51]. The expression of P-AKT and P-mTOR were both increased after treated with EXO from glioma cells under hypoxic condition and miR-25-3p overexpression in HMC3 and PBMCs-derived macrophage but abolished by miR-25-3p inhibitor and LY294002, an inhibitor of the PI3K-AKT signaling pathway (Fig. 4B-F). LY294002 also can significantly decrease the expression of ARG-1 and CD163 while increased the expression of TNF-α confirmed by western-blot (Fig. 4F), qRT-PCR and flow cytometry (Fig. 4G, H). Conformably, the increased proliferation and migration capacity of GBM#P3 caused by EXO-P3H were also abolished by LY294002 (Fig. 4I, J). These datas revealed that miR-25-3p enhances M2 polarization via PI3K-AKT pathways activation in microglial and macrophages.

### MiR-25-3p activated PI3K-AKT pathways by silencing PHLPP2

**Fig. 5:**
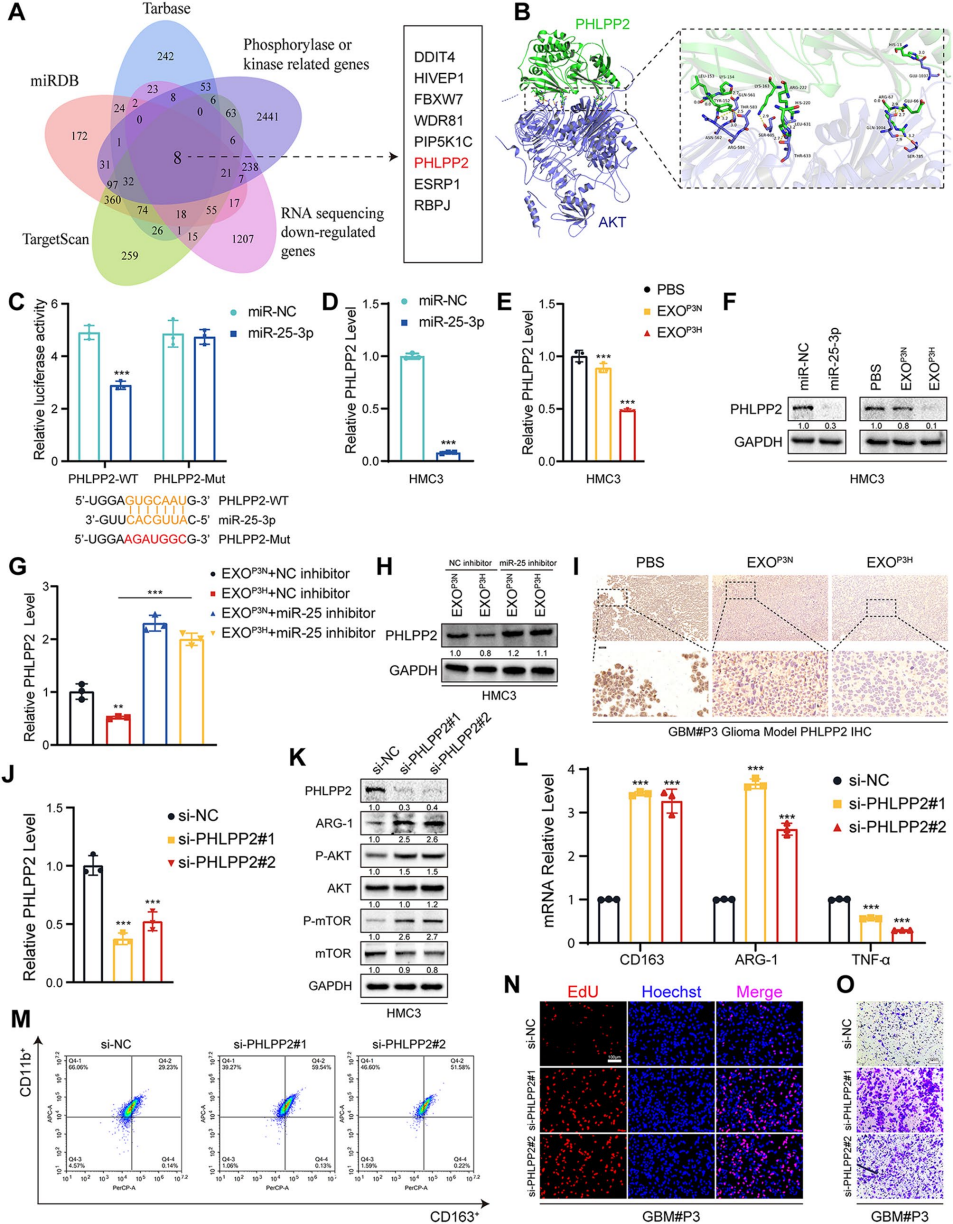
MiR-25-3p activated PI3K-AKT pathways by silencing PHLPP2. (**A**) The intersection of RNA sequencing down-regulated genes, Phosphorylase or kinase related genes and three microRNA databases were performed. (**B**) Molecular docking of PHLPP2 to AKT predicted with HDOCK. (**C**) Results of dual-luciferase reporter assay indicated that luciferase activity in 293T cells when miR-25-3p co-transfected with PHLPP2 wildtype or PHLPP2 mutant. Repeat three times for each sample. (**D**) qRT-PCR showed the mRNA expression levels of PHLPP2 in HMC3 transfected with miR-NC or miR-25-3p. Repeat three times for each sample. (**E**) qRT-PCR showed the mRNA expression levels of PHLPP2 in HMC3 treated with PBS, EXO^P3N^, and EXO^P3H^. Repeat three times for each sample. (**F**) Representative Western blot images and quantification of PHLPP2 levels in HMC3 treated as indicated. Repeat three times for each sample. (**G**) qRT-PCR showed the mRNA expression levels of PHLPP2 in HMC3 treated as indicated. The working concentration of miR-25 inhibitor for treating the cells was 0.06 µM. Repeat three times for each sample. (**H**) Representative Western blot images and quantification of PHLPP2 levels in HMC3 treated as indicated. The working concentration of miR-25 inhibitor for treating the cells was 0.06 µM. Repeat three times for each sample. (**I**) Representative images of IHC for PHLPP2 expression levels and quantification in sections from the indicated GBM#P3 xenograft. Scale bar = 100 μm. Repeat three times for each sample. (**J**) qRT-PCR showed the mRNA expression levels of PHLPP2 in HMC3 transfected with si-NC, si-PHLPP2#1 or si-PHLPP2#2. Repeat three times for each sample. (**K**) Representative Western blot images and quantification of PHLPP2, ARG-1, P-AKT, AKT, P-mTOR and mTOR in HMC3 transfected with si-NC, si-PHLPP2#1 or si-PHLPP2#2. Repeat three times for each sample. (**L**) qRT-PCR showed the mRNA expression levels of CD163, ARG-1 and TNF-α in HMC3 transfected with si-NC, si-PHLPP2#1 or si-PHLPP2#2. Repeat three times for each sample. (**M**) Flow cytometry to detect the proportion of CD163 positive HMC3 transfected with si-NC, si-PHLPP2#1 or si-PHLPP2#2. Repeat three times for each sample. (**N**) EdU assay was used to evaluate the proliferation of GBM#P3 co-cultured with HMC3 transfected with si-NC, si-PHLPP2#1 or si-PHLPP2#2. Scale bar = 100 μm. Repeat three times for each sample. (**O**) The migratory capacity of GBM#P3 co-cultured with HMC3 transfected with si-NC, si-PHLPP2#1 or si-PHLPP2#2. Scale bar = 100 μm. Repeat three times for each sample. Data are shown as mean ± SD. *P < 0.05, **P < 0.01, ***P < 0.001

While we have established that miR-25-3p from hypoxic glioblastoma sources can induce macrophage M2 polarization through the activation of the PI3K-AKT signaling pathway, the precise mechanisms by which miR-25-3p activates this pathway remain unclear. To address this, we intersected the database-predicted downstream targets of miR-25-3p with genes downregulated in RNA-seq and genes associated with phosphorylation. This analysis identified eight genes, including DDIT4, HIVEP1, FBXW7, WDR81, PIP5K1C, PHLPP2, ESRP1, and RBPJ (Fig. 5A). Through the target score of miRDB and protein-protein interaction (PPI) networks and the scoring of miR-25-3p downstream targets, we discovered that PHLPP2 may be the most closely linked target to miR-25-3p (Figure S6A, B). Using HDOCK protein docking, we identified the corresponding binding sites between PHLPP2 and AKT (Fig. 5B), and previous research has indicated that PHLPP2 is a critical protein involved in the dephosphorylation of AKT [52]. Furthermore, through analysis of the TCGA database, we observed that PHLPP2 is downregulated in GBM (Figure S6C). The reduced expression of PHLPP2 is significantly associated with unfavorable prognosis in glioblastoma patients (Figure S6D-E). Therefore, PHLPP2 has been selected as a downstream target. To establish whether PHLPP2 are targets of miR-25-3p, 3′ UTRs of PHLPP2 were cloned into the luciferase plasmid PmirGLO in HEK293T. Notably, the luciferase activities of 3′ UTR of PHLPP2 were suppressed by miR-25-3p, while this effect was abolished by mutant 3′ UTR of PHLPP2 (Fig. 5C). Moreover, the expression of PHLPP2 was inhibited by overexpression of miR-25-3p (Fig. 5D, Figure S7A). The relative PHLPP2 levels were similarly decreased by the addition of normoxic and hypoxic glioma exosomes. While being treated with miR-25-3p inhibitor, the relative PHLPP2 levels were increased as expected (Fig. 5E-H, Figure S7B-G). Similarly, in vivo experiments, IHC staining of mouse brain tissue slices treated with EXO-P3H revealed a reduction in the expression level of PHLPP2 (Fig. 5I, Figure S7H). To validate the function of PHLPP2, we performed PHLPP2 knockdown experiments in HMC3 cells (Fig. 5J, K). We observed that PHLPP2 knockdown promoted the activation of the PI3K-AKT signaling pathway, resulting in increased expression levels of ARG-1 and CD163, along with a decrease in TNF-α expression (Fig. 5K-M, Figure S7I). As expected, when GBM#P3 co-cultured with HMC3 with si-NC, si-PHLPP2#1 or si-PHLPP2#2, knockdown PHLPP2 significantly promoted the proliferation and migration of GBM#P3 (Fig. 5N, O, Figure S7J, K) These results revealed that miR-25-3p activated PI3K-AKT pathway by silencing PHLPP2.

### Knockdown of mir-25-3p inhibits the GBM malignant progression

**Fig. 6:**
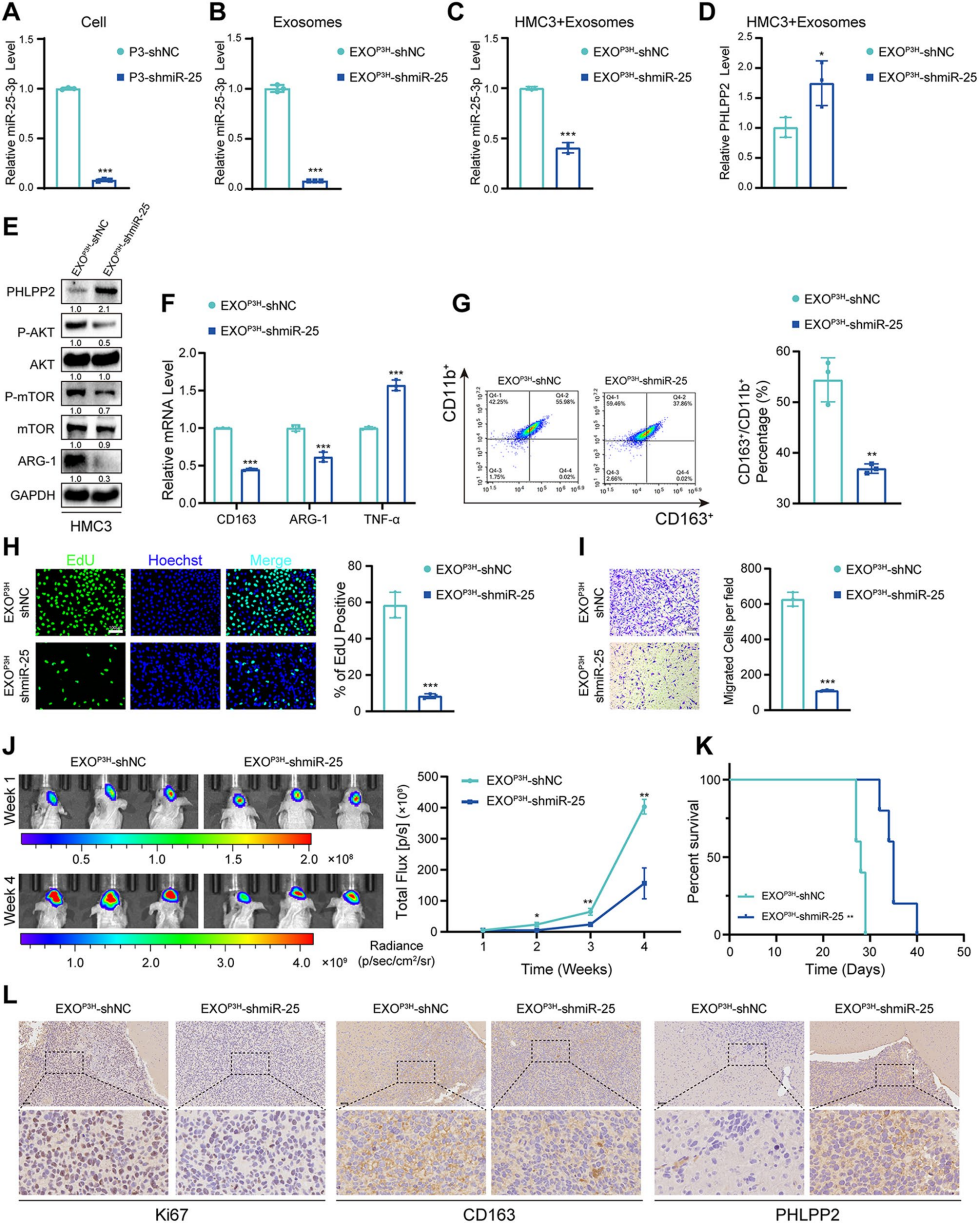
Knockdown of miR-25-3p inhibits the GBM malignant progression. (**A**) qRT-PCR showed the relative miR-25-3p level in GBM#P3 with miR-25-3p knockdown. Repeat three times for each sample. (**B**) qRT-PCR showed the relative miR-25-3p level in exosomes derived by GBM#P3 with miR-25-3p knockdown. Repeat three times for each sample. (**C**) qRT-PCR showed the relative miR-25-3p level in HMC3 treated with exosomes derived by GBM#P3 under hypoxic condition with miR-NC (EXO^P3H^-shNC) or miR-25-3p knockdown (EXO^P3H^-shmiR-25). Repeat three times for each sample. (**D**) qRT-PCR showed the relative PHLPP2 level in HMC3 treated with exosomes derived by GBM#P3 under hypoxic condition with miR-NC (EXOP3H-shNC) or miR-25-3p knockdown (EXOP3H-shmiR-25). Repeat three times for each sample. (**E**) Representative Western blot images and quantification of PHLPP2, P-AKT, AKT, P-mTOR and mTOR in HMC3 treated with exosomes derived by GBM#P3 under hypoxic condition with miR-NC (EXOP3H-shNC) or miR-25-3p knockdown (EXOP3H-shmiR-25). Repeat three times for each sample. (**F**) qRT-PCR showed the mRNA expression levels of CD163, ARG-1 and TNF-α in HMC3 treated with exosomes derived by GBM#P3 under hypoxic condition with miR-NC (EXOP3H-shNC) or miR-25-3p knockdown (EXOP3H-shmiR-25). Repeat three times for each sample. (**G**) Flow cytometry to detect the proportion of CD163 positive HMC3 treated with exosomes derived by GBM#P3 under hypoxic condition with miR-NC (EXOP3H-shNC) or miR-25-3p knockdown (EXOP3H-shmiR-25). Repeat three times for each sample. (**H**) EdU assay was used to evaluate the proliferation of GBM#P3 co-cultured with HMC3 treated with exosomes derived by GBM#P3 under hypoxic condition with miR-NC (EXOP3H-shNC) or miR-25-3p knockdown (EXOP3H-shmiR-25). Scale bar = 100 μm. Repeat three times for each sample. (**I**) The migratory capacity of GBM#P3 co-cultured with HMC3 t treated with exosomes derived by GBM#P3 under hypoxic condition with miR-NC (EXOP3H-shNC) or miR-25-3p knockdown (EXOP3H-shmiR-25). Scale bar = 100 μm. Repeat three times for each sample. (**J**) Bioluminescence imaging and quantification of tumors derived from GBM#P3 luciferase shNC or shmiR-25-3p and HMC3 orthotopically implanted into mice. Representative images on Week1 and Week 4 post-implantation are shown. (**K**) Kaplan-Meier survival curve for mice with GBM#P3-shNC, -shmiR-25-3p derived xenografts. The Log-rank test was performed to gain statistical significance. (**L**) Representative images of IHC for Ki67, CD163 and PHLPP2 expression levels and quantification in sections from the indicated GBM#P3 xenograft. Scale bar = 100 μm. Repeat three times for each sample. Data are shown as mean ± SD. *P < 0.05, **P < 0.01, ***P < 0.001

To further investigate the impact of miR-25-3p on microglial and macrophage M2 polarization in vivo, we established a stable GBM#P3 cell line with miR-25-3p knockdown, and qRT-PCR analysis confirmed a significant reduction in miR-25-3p levels in both the cells and their exosomes (Fig. 6A, B). When exosomes derived from GBM#P3-shNC (EXO^P3H^-shNC) and GBM#P3-shmiR-25-3p (EXO^P3H^-shmiR-25) under hypoxic condition were co-cultured with HMC3, it was observed that the levels of PHLPP2 expression significantly increased in HMC3 cells treated with EXO^P3H^-shmiR-25 compared to those treated with EXO^P3H^-shNC (Fig. 6C, D). Moreover, the phosphorylation of AKT and mTOR were inhibited with miR-25-3p knockdown (Fig. 6E). So, the expression of M2-like phenotype marker CD163 and ARG-1 were inhibited in the group treated with EXO^P3H^-shmiR-25 (Fig. 6E-G).

Consistently, the proliferation and migration of GBM#P3 were also decreased when co-cultured with HMC3 treated with EXO^P3H^-shmiR-25 (Fig. 6H, I). However, when we conducted rescue experiments by administering SC79 (a phosphorylated AKT activator) in the EXO^P3H^-shmiR-25 treated group, the phenotypic changes caused by miR-25 knockdown were successfully reversed (Figure S8A-E). In vivo bioluminescent imaging showed that nude mice implanted with miR-25-3p knockdown glioma cells had weaker bioluminescence signals and longer survival time (median survival time = 35 days) than those in the control group (median survival time = 28 days) (Fig. 6J, K). IHC staining also revealed lower expression levels of Ki67 and CD163 and higher expression levels of PHLPP2 in the brains of mice with orthotopically implanted GBM#P3-shmiR-25-3p (Fig. 6L, Figure S8F).

Therefore, the results demonstrated that knockdown of miR-25-3p in glioma cells inhibits glioma malignant progression in vitro and in vivo.

## Discussion

Glioblastoma is the most malignant primary intracranial tumor, and it constitutes a highly intricate ecosystem, encompassing tumor cells, astrocytes, vascular endothelial cells, immune cells, and other microenvironmental cells. Previous research has indicated that the crosstalk and communication between glioblastoma cells and macrophages lead to sustained tumor proliferation, invasion, angiogenesis, and drug resistance [53–56]. It is not limited to glioblastoma; immune-suppressive microenvironments exist in many tumors. The presence of immune-suppressive microenvironments contributes to the clinical failure of therapeutic interventions in numerous cancer types [57, 58]. Furthermore, certain primary tumors have the ability to reshape the systemic immune microenvironment, resulting in immune-suppressed conditions within target organs, creating a conducive ‘soil’ for tumor growth. This, in turn, leads to the metastasis of primary tumors and significantly impacts patient prognosis [59–62].

However, the mechanisms underlying the formation of immune-suppressive microenvironments remain elusive. It is uncertain whether tumors reshape the microenvironment or if changes in the patient’s immune microenvironment occur due to genetic alterations or environmental factors. This alteration may result in a lack of immune surveillance over aberrantly proliferating cells, ultimately leading to tumorigenesis. Currently, there is no definitive answer to this question. Previous studies have indicated that tumor cells can influence immune cells and reshape the immune microenvironment through various mechanisms. This includes direct modification of immune cells through cell surface receptor engagement [63, 64], as well as the release of cytokines, secreted proteins, and other factors that act on immune cell receptors [65, 66], resulting in alterations in immune cell phenotypes. Due to the complexity of the tumor microenvironment, many factors, including cytokines, secreted proteins, lipids, nucleic acids, and more, released by tumors are susceptible to rapid degradation, particularly when reshaping distant organ immune microenvironments.

Exosomes, on the other hand, are extracellular vesicles enclosed by lipid membranes and contain a rich cargo of proteins, lipids, nucleic acids, and other molecules [15, 67]. Their membranous structure provides effective protection for the enclosed contents against enzymatic degradation within the body, allowing them to serve as a robust medium for communication and signaling between tumor cells and other cells.

Our study demonstrated that glioblastoma cells, particularly under hypoxic condition, release exosomes that stimulate microglia and macrophage M2 polarization. M2-polarized microglia and macrophage, in turn, secrete immunosuppressive cytokines such as IL-8 and IL-10, VEGF, RANTES, MCP-3, GRO-α creating an immunosuppressive microenvironment that fosters glioblastoma progression. For the tumor, this sets up a benign positive feedback loop. Our model simulated that EVs from glioma cells in a hypoxic zone of the tumor that can also reach normoxic areas of the tumor and affect the macrophages or microglia there. In this process, miR-25-3p within glioblastoma-derived exosomes plays a pivotal role. miR-25-3p can be readily delivered to macrophages via exosomes. Once inside macrophages, miR-25-3p binds to the 3’UTR region of PHLPP2 mRNA, leading to enhanced mRNA degradation. Consequently, the synthesis of PHLPP2 protein decreases. Since PHLPP2 is a crucial dephosphorylating enzyme, it leads to the dephosphorylation of AKT. Our previous research indicates that the phosphorylation activation of the AKT signaling pathway enhances tumor proliferation, invasion, and migration [68]. Additionally, findings from other researchers suggest that AKT signaling pathway phosphorylation activation leads to macrophage M2 polarization, providing robust support for our conclusions [31, 51].

While the communication and crosstalk between tumor cells and immune cells lead to the immunosuppressive microenvironment, posing significant challenges in glioblastoma therapy, it also harbors numerous opportunities. Many studies have demonstrated that external interventions can reshape the tumor microenvironment into an immunocompetent microenvironment. In comparison to direct pharmacological cytotoxicity against tumor cells, immunotherapy harnesses internal resources more effectively, mobilizing the immune surveillance and cytotoxic capabilities of the host’s immune cells. This offers substantial benefits for patient prognosis [69–71]. This study has some limitations, as we have not identified effective drugs capable of crossing the blood-brain barrier to reshape the immunosuppressive microenvironment and subsequently target glioblastoma cells. However, our research has revealed miR-25-3p as a risk factor for glioblastoma patients, indicating its involvement in shaping the immunosuppressive microenvironment. Targeted therapies directed at miR-25-3p and the PI3K-AKT-mTOR signaling pathway may disrupt the immunosuppressive microenvironment and play a role in tumor immunotherapy. This offers new avenues for immunotherapy in glioblastoma. In future research, we will explore the potential of using mediators like exosomes to deliver inhibitors of miR-25-3p, along with drugs that can inhibit microglia and macrophage M2 polarization, aiming to counteract the immunosuppressive effects of miR-25-3p.

## Conclusion

In this study, we uncovered miR-25-3p, originating from hypoxic glioma cells, is transported to macrophages through exosomes, serving as a mediator that facilitate M2 polarization in macrophages via the miR-25-3p/PHLPP2/PI3K-AKT-mTOR signaling pathway. Our findings suggested that strategies aimed at regulating miR-25-3p levels, its transmission, or the inhibition of the PI3K-AKT-mTOR pathway’s activation may interfere with the immunosuppressive milieu, offering an innovative strategy for glioma immunotherapy.

## Electronic supplementary material

Below is the link to the electronic supplementary material.

Supplementary Material 1

## Data Availability

No datasets were generated or analysed during the current study.

## Abbreviations

ATCC: American Type Culture Collection
BSA: Bovine Serum Albumin
bFGF: Basic Fibroblast Growth Factor
CNS: Central Nervous System
EGF: Epidermal Growth Factor
EMEM: Eagle’s Minimum Essential Medium
EVs: Extracellular Vesicles
EXO: Exosome
EXO^P3H^: Exosomes from Hypoxic P3 cells
EXO^P3N^: Exosomes from Normoxic P3 cells
EXO^T98H^: Exosomes from Hypoxic T98 cells
EXO^T98N^: Exosomes from Normoxic T98 cells
EXO^U251H^: Exosomes from Hypoxic U251 cells
EXO^U251N^: Exosomes from Normoxic U251 cells
EXO^GL261H^: Exosomes from Hypoxic GL261 cells
EXO^GL261N^: Exosomes from Normoxic GL261 cells
FBS: Fetal Bovine Serum
GBM: Glioblastoma
GSCs: GBM Stem-like Cells
HMC3: Human Microglial Cell line
IHC: Immunohistochemistry
NTA: Nanoparticle Tracking Analysis
TEM: Transmission Electron Microscopy
TME: Tumor Microenvironment
PBMCs: Peripheral blood mononuclear cells
PBS: Phosphate Buffer Saline
PFA: Paraformaldehyde
