# Supplementary material for: Hypoxic glioma-derived exosomal miR-25-3p promotes macrophage M2 polarization by activating the PI3K-AKT-mTOR signaling pathway
Source: J Nanobiotechnology. 2024 Oct 16;22:628. doi: 10.1186/s12951-024-02888-5 (PMC11481566; doi:10.1186/s12951-024-02888-5)
Supplement: Supplementary file 1 — Supplementary Material 1 [file 12951_2024_2888_MOESM1_ESM.docx]

**Supplementary Information**

# Hypoxic glioma-derived exosomal miR-25-3p promotes macrophage M2 polarization by activating the PI3K-AKT-mTOR signaling pathway

## Author name and affiliations:

Zhiwei Xue^1*^, Junzhi Liu^1*^, Wenchen Xing^1^, Feiyu Mu^1^, Yanzhao Wu^1^, Jiangli Zhao^1^, Xuchen Liu^1^, Donghai Wang^1, 3^, Jian Wang^1, 4^, Xingang Li^1, 2#^, Jiwei Wang^1#^, Bin Huang^1, 2, 3#^

1.Department of Neurosurgery, Qilu Hospital, Cheeloo College of Medicine and Institute of Brain and Brain-Inspired Science, Shandong University, Jinan, China.

2. Jinan Microecological Biomedicine Shandong Laboratory and Shandong Key Laboratory of Brain Function Remodeling, Jinan, China.

3. Department of Neurosurgery, Qilu Hospital of Shandong University Dezhou Hospital, Dezhou, China

4. Department of Biomedicine, University of Bergen, Jonas Lies Vei 91, 5009, Bergen, Norway.

* Contributed equally to this work

## #Corresponding Author:

Bin Huang, Ph.D. (hb@sdu.edu.cn)

Jiwei Wang, Ph.D. (Jiwei.wang@email.sdu.edu.cn)

Xingang Li, Ph.D. and M.D. ([lixg@sdu.edu.cn](mailto:lixg@sdu.edu.cn))

This supplementary information contains:

- 23 Pages
- Supplementary Figures (8 Figures, S1 to S8)
- Supplementary Tables (6 Tables, S1 to S6)

## Supplementary Figures

### Figure S1


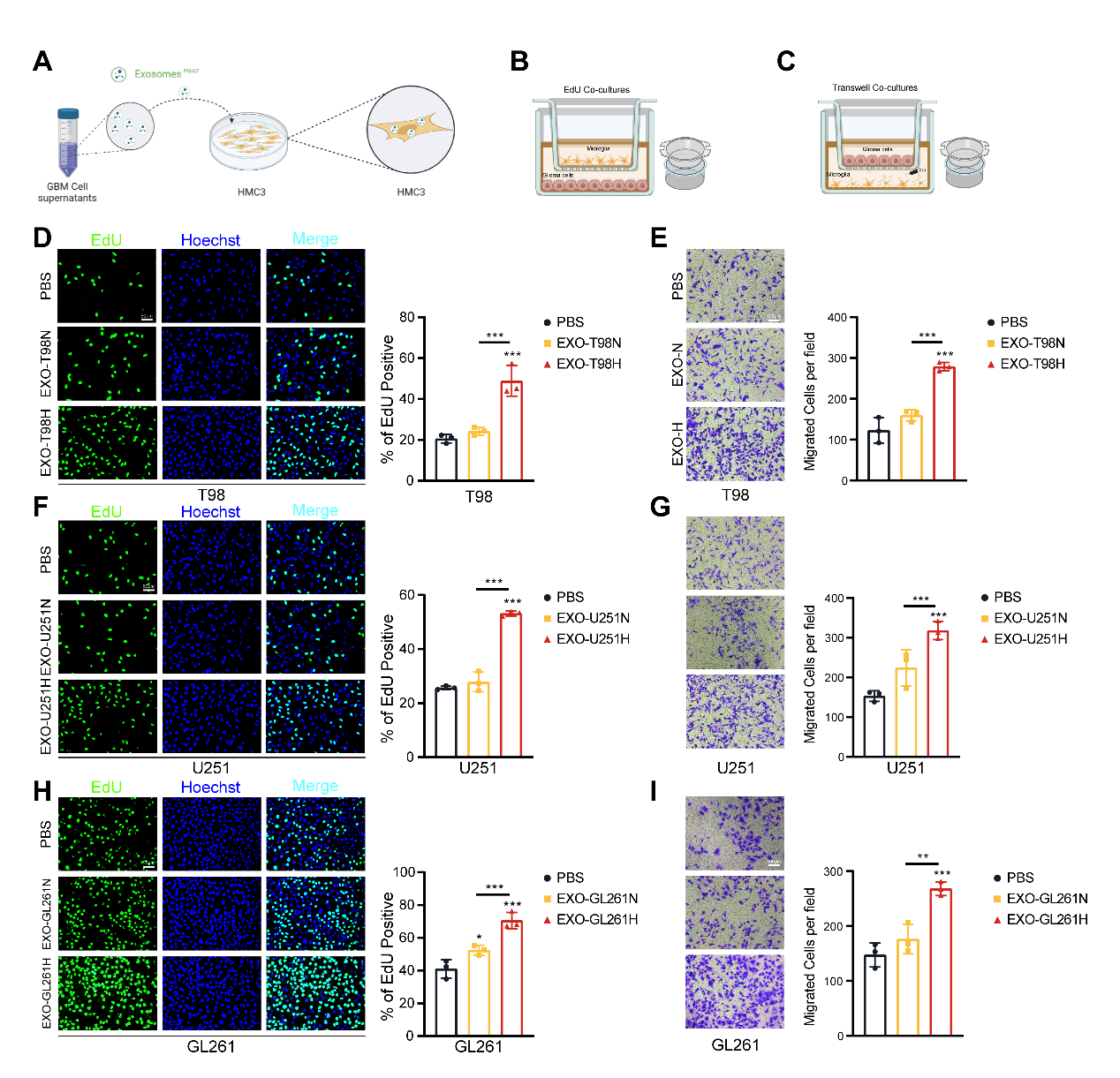


(A) Schematic diagram of how exosomes were extracted, added to cell culture medium and uptaken by cells.

(B) Schematic diagram of EdU assay.

(C) Schematic diagram of transwell assay.

(D) EdU assay was used to evaluated the proliferation of T98 co-cultured with HMC3 treated by PBS, EXO-T98N or EXO-T98H. Repeat three times for each sample. The results were quantified. Scale bar = 50 μm.

(E) The migratory capacity of T98 co-cultured with HMC3 treated by PBS, EXO-T98N or EXO-T98H. Repeat three times for each sample. The results were quantified. Scale bar = 100 μm.

(F) EdU assay was used to evaluated the proliferation of U251 co-cultured with HMC3 treated by PBS, EXO-U251N or EXO-U251H. Repeat three times for each sample. The results were quantified. Scale bar = 50 μm.

(G) The migratory capacity of U251 co-cultured with HMC3 treated by PBS, EXO-U251N or EXO-U251H. Repeat three times for each sample. The results were quantified. Scale bar = 100 μm.

(H) EdU assay was used to evaluated the proliferation of GL261 co-cultured with RAW264.7 treated by PBS, EXO-GL261N or EXO-GL261H. Repeat three times for each sample. The results were quantified. Scale bar = 50 μm.

(I) The migratory capacity of GL261 co-cultured with RAW264.7 treated by PBS, EXO-GL261N or EXO-GL261H. Repeat three times for each sample. The results were quantified. Scale bar = 100 μm.

Data are shown as mean ± SD. **P* < 0.05, ***P* < 0.01, ****P* < 0.001.

### Figure S2


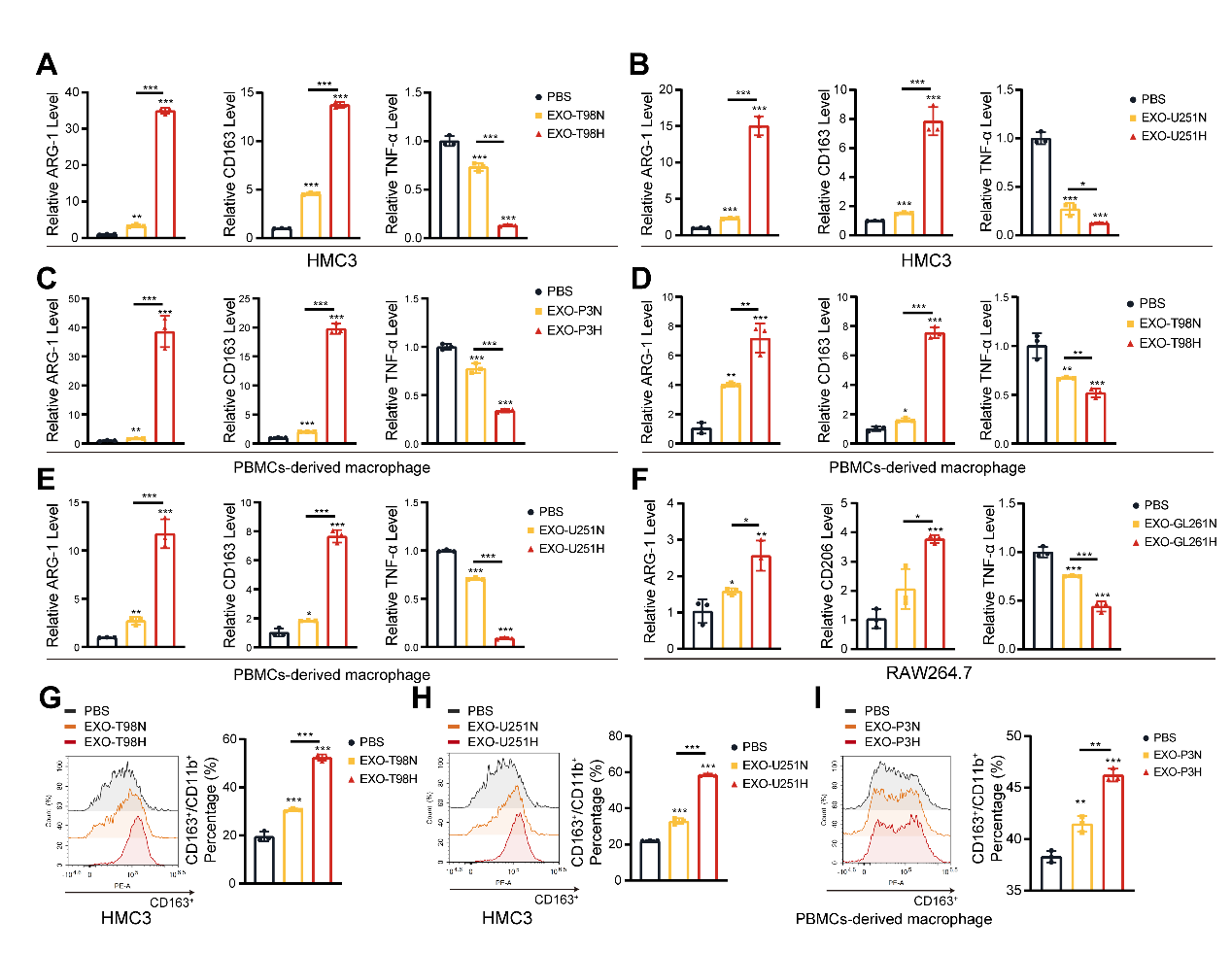


(A) qRT-PCR showed the mRNA expression levels of ARG-1, CD163 and TNF-α in HMC3 treated with PBS, EXO-T98N, and EXO-T98H 48h. Repeat three times for each sample.

(B) qRT-PCR showed the mRNA expression levels of ARG-1, CD163 and TNF-α in HMC3 treated with PBS, EXO-U251N, and EXO-U251H 48h. Repeat three times for each sample.

(C) qRT-PCR showed the mRNA expression levels of ARG-1, CD163 and TNF-α in PBMCs-derived macrophage treated with PBS, EXO-P3N, and EXO-P3H 48h. Repeat three times for each sample.

(D) qRT-PCR showed the mRNA expression levels of ARG-1, CD163 and TNF-α in PBMCs-derived macrophage treated with PBS, EXO-T98N, and EXO-T98H 48h. Repeat three times for each sample.

(E) qRT-PCR showed the mRNA expression levels of ARG-1, CD163 and TNF-α in PBMCs-derived macrophage treated with PBS, EXO-U251N, and EXO-U251H 48h. Repeat three times for each sample.

(F) qRT-PCR showed the mRNA expression levels of ARG-1, CD163 and TNF-α in RAW264.7 treated with PBS, EXO-GL261N, and EXO-GL261H 48h. Repeat three times for each sample.

(G) Flow cytometry to detect the proportion of CD163 positive HMC3 following treatment with PBS, EXO-T98N, and EXO-T98H 48h. Repeat three times for each sample.

(H) Flow cytometry to detect the proportion of CD163 positive HMC3 following treatment with PBS, EXO-U251N, and EXO-U251H 48h. Repeat three times for each sample.

(I) Flow cytometry to detect the proportion of CD163 positive PBMCs-derived macrophage following treatment with PBS, EXO-P3N, and EXO-P3H 48h. Repeat three times for each sample.

Data are shown as mean ± SD. **P* < 0.05, ***P* < 0.01, ****P* < 0.001.

### Figure S3


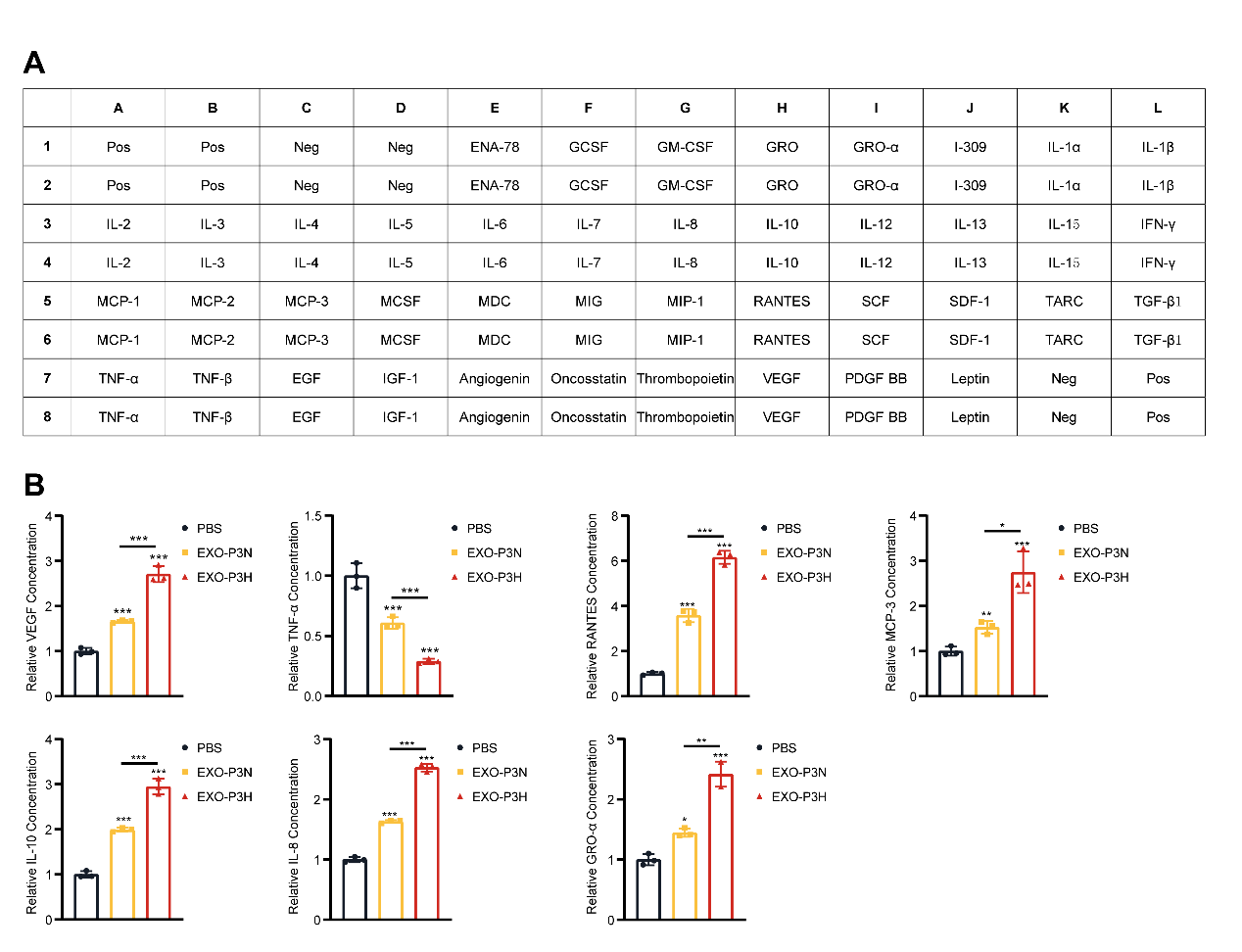


(A) The indication of the Human Cytokine Antibody Array (Abcam, Cambridge, MA, USA) plate plan.

(B) ELISA to determine the concentration of VEGF, TNF-α, RANTES, MCP-3, IL-10, IL-8 and GRO-α in supernatants from HMC3 treated with PBS, EXO-P3N or EXO-P3H. Repeat three times for each sample.

Data are shown as mean ± SD. **P* < 0.05, ***P* < 0.01, ****P* < 0.001.

### Figure S4


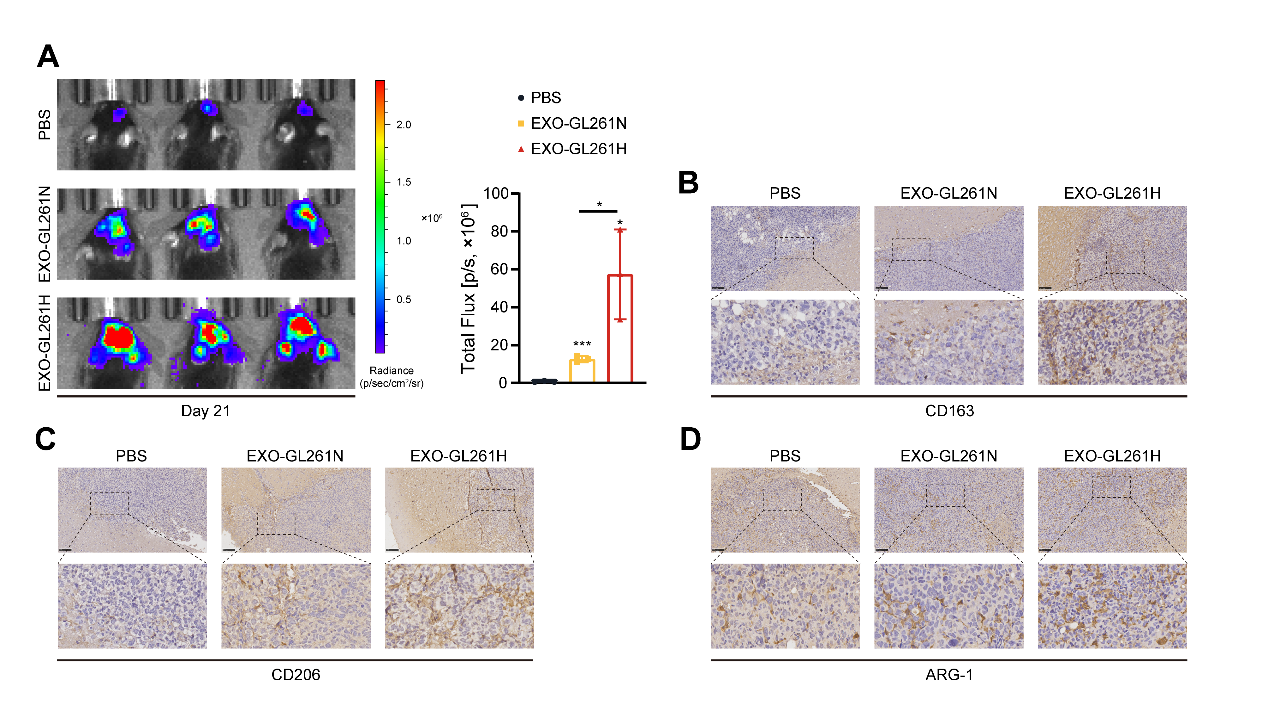


(A) Bioluminescence imaging and quantification of tumors derived from GL261 luciferase orthotopically implanted into mice treated as indicated. Representative images on day 21 post-implantation are shown.

(B) Representative images of IHC for CD163 expression levels in sections from the indicated GL261 xenograft. Scale bar = 100 µm. Repeat three times for each sample.

(C) Representative images of IHC for CD206 expression levels in sections from the indicated GL261 xenograft. Scale bar = 100 µm. Repeat three times for each sample.

(D) Representative images of IHC for ARG-1 expression levels in sections from the indicated GL261 xenograft. Scale bar = 100 µm. Repeat three times for each sample.

Data are shown as mean ± SD. **P* < 0.05, ***P* < 0.01, ****P* < 0.001.

### Figure S5


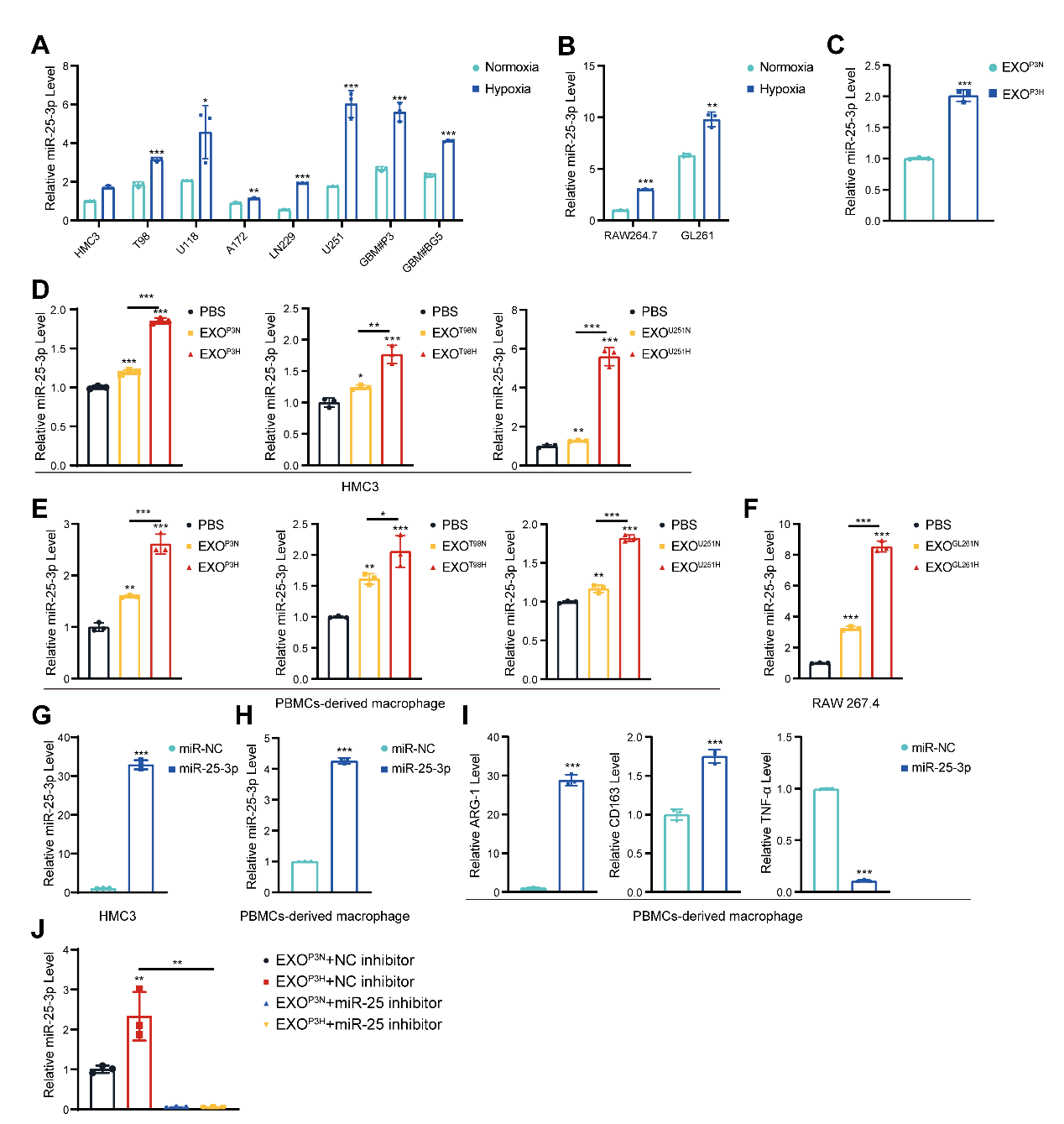


(A) qRT-PCR showed the expression levels of miR-25-3p in different glioma cell lines and HMC3 under hypoxic and normoxic conditions. Repeat three times for each sample.

(B) qRT-PCR showed the expression levels of miR-25-3p in RAW264.7 and GL261 under hypoxic and normoxic conditions. Repeat three times for each sample.

(C) qRT-PCR showed the expression levels of miR-25-3p in EXO from GBM#P3 under nomoxic or hypoxic condition. Repeat three times for each sample.

(D) qRT-PCR showed the expression levels of miR-25-3p in HMC3 treated as indicated 48h. Repeat three times for each sample.

(E) qRT-PCR showed the expression levels of miR-25-3p in PBMCs-derived macrophage treated as indicated 48h. Repeat three times for each sample.

(F) qRT-PCR showed the expression levels of miR-25-3p in RAW264.7 treated as indicated 48h. Repeat three times for each sample.

(G) qRT-PCR showed the expression levels of miR-25-3p in HMC3 transfected with miR-NC or miR-25-3p 48h. Repeat three times for each sample.

(H) qRT-PCR showed the expression levels of miR-25-3p in PBMCs-derived macrophage transfected with miR-NC or miR-25-3p 48h. Repeat three times for each sample.

(I) qRT-PCR showed the expression levels of CD163, ARG-1 and TNF-α in PBMCs-derived macrophage transfected with miR-NC or miR-25-3p 48h. Repeat three times for each sample.

(J) qRT-PCR showed the expression levels of miR-25-3p in HMC3 treated as indicated 48h. The working concentration of miR-25 inhibitor for treating the cells was 0.06μM. Repeat three times for each sample.

Data are shown as mean ± SD. **P* < 0.05, ***P* < 0.01, ****P* < 0.001.

### Figure S6


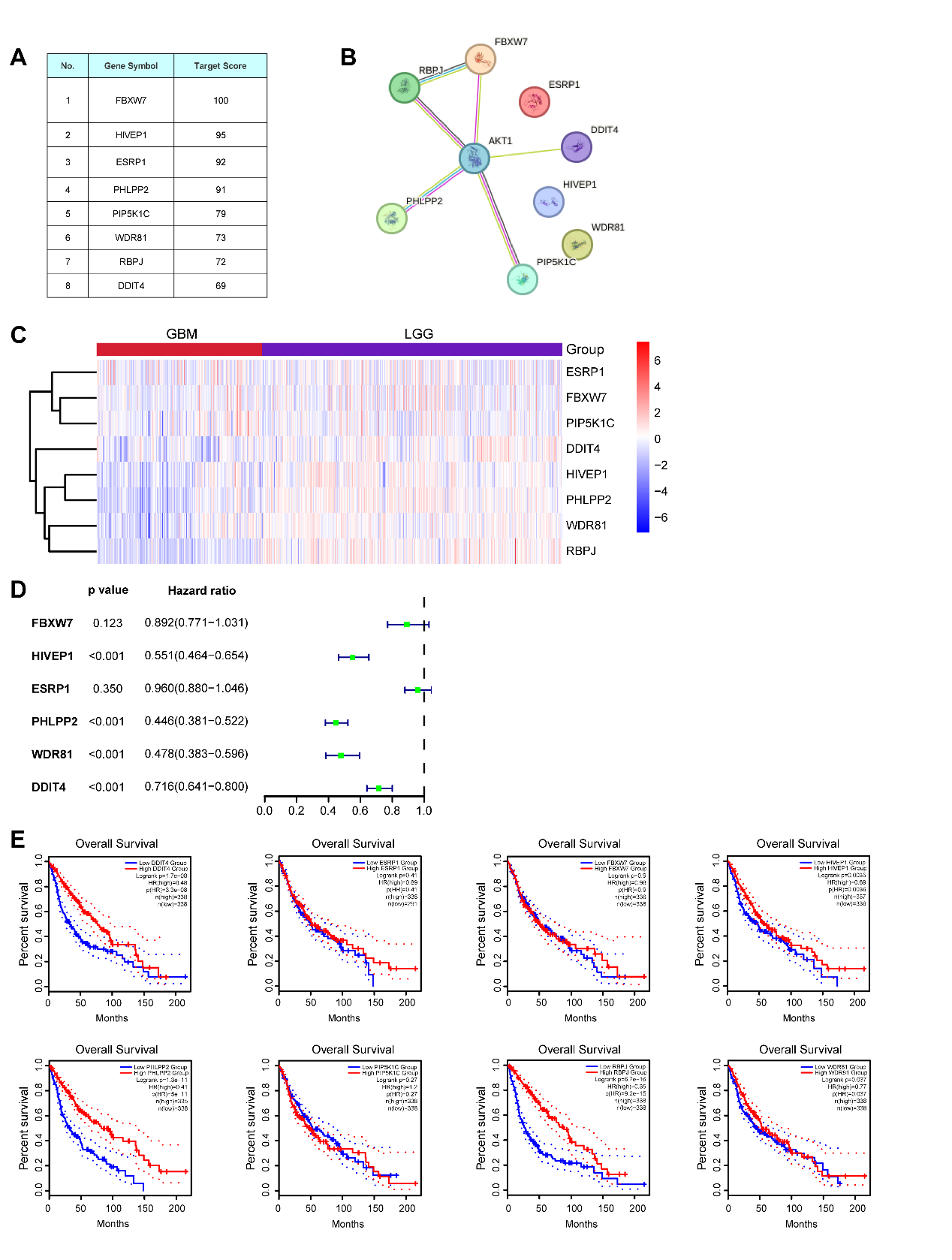


(A) The target score of the 3'UTR region of the 8 intersecting genes obtained from miRDB (https://mirdb.org/) with miR-25-3p.

(B) STRING (string-db.org) analysis of AKT, PHLPP2, RBPJ, FBXW7, ESRP1, DDIT4, HIVEP1, WDR81, and PIP5K1C protein interaction network.

(C) Heatmap of the expression of ESRP1, FBXW7, PIP5K1C, DDIT4, HIVEP1, PHLPP2, WDR81, and RBPJ in GBM and LGG from the TCGA database.

(D) Univariate analyses for FBXW7, HIVEP1, ESRP1, PHLPP2, WDR81, and DDIT4 of overall survival in the TCGA database.

(E) Kaplan-Meier survival curves of patients with high and low expression of ESRP1, FBXW7, PIP5K1C, DDIT4, HIVEP1, PHLPP2, WDR81, and RBPJ based on the TCGA database.

Data are shown as mean ± SD. **P* < 0.05, ***P* < 0.01, ****P* < 0.001.

### Figure S7


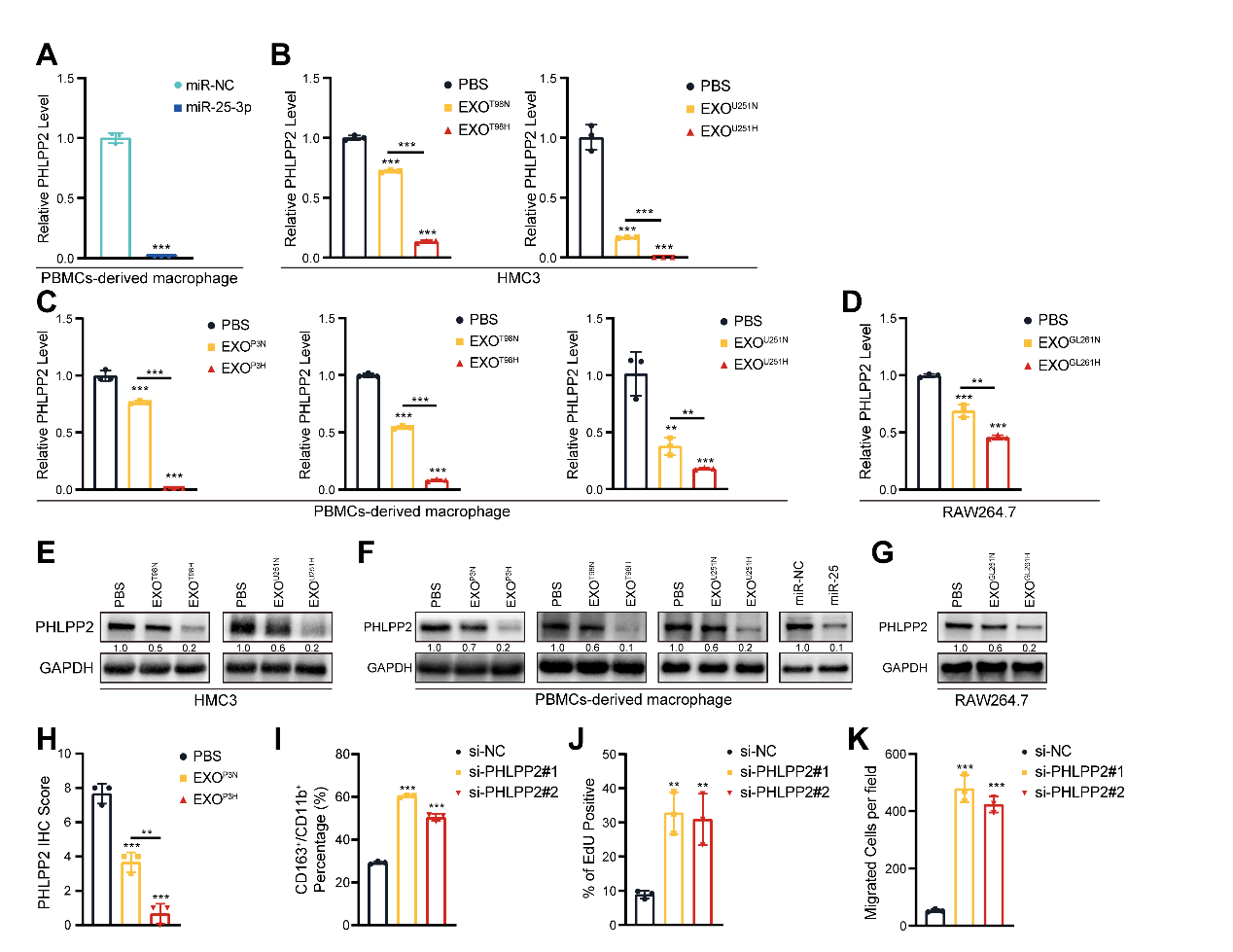


(A) qRT-PCR showed the mRNA expression levels of PHLPP2 in PBMCs-derived macrophage transfected with miR-NC or miR-25-3p 48h. Repeat three times for each sample.

(B) qRT-PCR showed the mRNA expression levels of PHLPP2 in HMC3 treated as indicated 48h. Repeat three times for each sample.

(C) qRT-PCR showed the mRNA expression levels of PHLPP2 in PBMCs-derived macrophage treated as indicated 48h. Repeat three times for each sample.

(D) qRT-PCR showed the mRNA expression levels of PHLPP2 in RAW264.7 treated as indicated 48h. Repeat three times for each sample.

(E) Representative Western blot images and quantification of PHLPP2 levels in HMC3 treated as indicated. Repeat three times for each sample.

(F) Representative Western blot images and quantification of PHLPP2 levels in PBMCs-derived macrophage treated as indicated. Repeat three times for each sample.

(G) Representative Western blot images and quantification of PHLPP2 levels in RAW264.7 treated as indicated. Repeat three times for each sample.

(H) Quantification of PHLPP2 IHC score in sections from the indicated GBM#P3 xenograft. Repeat three times for each sample.

(I) Quantification of CD163 and CD11b positive cells in HMC3 transfected with si-NC, si-PHLPP2#1 or si-PHLPP2#2. Repeat three times for each sample.

(J) Quantification of EdU positive rate of GBM#P3 co-cultured with HMC3 transfected with si-NC, si-PHLPP2#1 or si-PHLPP2#2. Repeat three times for each sample.

(K) Quantification of migrated GBM#P3 cells co-cultured with HMC3 transfected with si-NC, si-PHLPP2#1 or si-PHLPP2#2. Repeat three times for each sample.

Data are shown as mean ± SD. **P* < 0.05, ***P* < 0.01, ****P* < 0.001.

### Figure S8


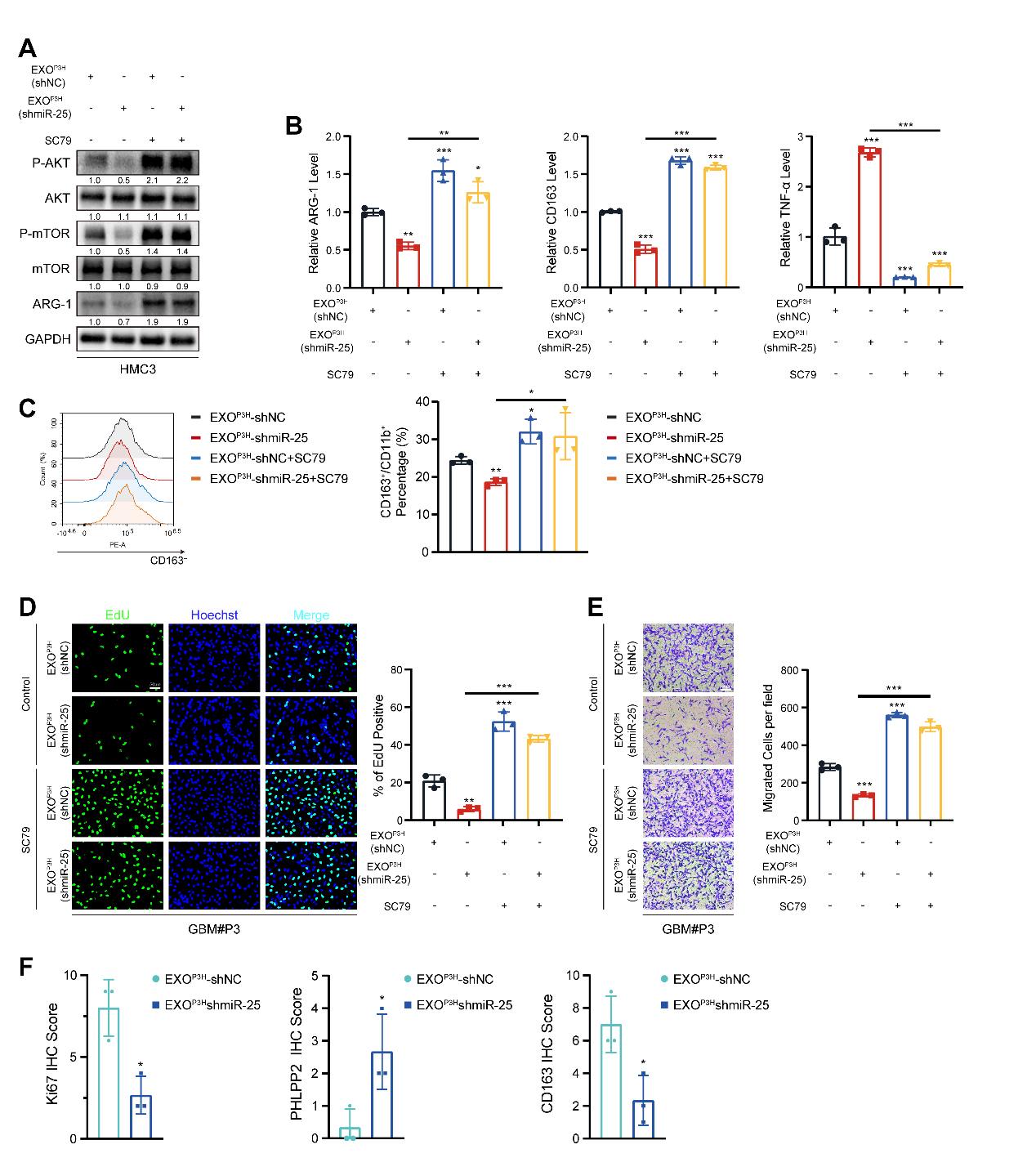


(A) Representative Western blot images and quantification of PHLPP2, P-AKT, AKT, P-mTOR, mTOR and ARG-1 levels in HMC3 treated as indicated. Repeat three times for each sample.

(B) qRT-PCR showed the mRNA expression levels of ARG-1, CD163 and TNF-α in HMC3 treated as indicated. Repeat three times for each sample.

(C) Flow cytometry to detect the proportion of CD163 positive HMC3 following treatment as indicated. Repeat three times for each sample.

(D) EdU assay was used to evaluated the proliferation of GBM#P3 co-cultured with HMC3 treated as indicated. The results were quantified. Scale bar = 50μm. Repeat three times for each sample.

(E) The migrated capacity of GBM#P3 co-cultured with HMC3 treated as indicated. The results were quantified. Scale bar = 100 μm. Repeat three times for each sample.

(F) Quantification of Ki67, PHLPP2, and CD163 IHC score in sections from the indicated GBM#P3 xenograft. Repeat three times for each sample.

Data are shown as mean ± SD. **P* < 0.05, ***P* < 0.01, ****P* < 0.001.

## Supplementary Tables

### Table S1 Clinical data of glioma patients involved in our study

| Patient ID | Age at  diagnosis | Gender | WHO Grade | IDH |
| --- | --- | --- | --- | --- |
| 21158770 | 31 | Female | 2 | Mut |
| 21213163 | 58 | Female | 2 | Mut |
| 23083442 | 53 | Male | 2 | Mut |
| 23071004 | 34 | Female | 3 | Mut |
| 22003744 | 31 | Female | 3 | Mut |
| 21209440 | 56 | Male | 3 | Mut |
| 21157504 | 61 | Female | 4 | WT |
| 21218013 | 56 | Female | 4 | WT |
| 22045943 | 80 | Male | 4 | WT |
| 22000465 | 46 | Female | 4 | WT |
| 22147580 | 53 | Male | 4 | WT |

### Table S2. STR profiles of GBM cells in this study

GBM#P3


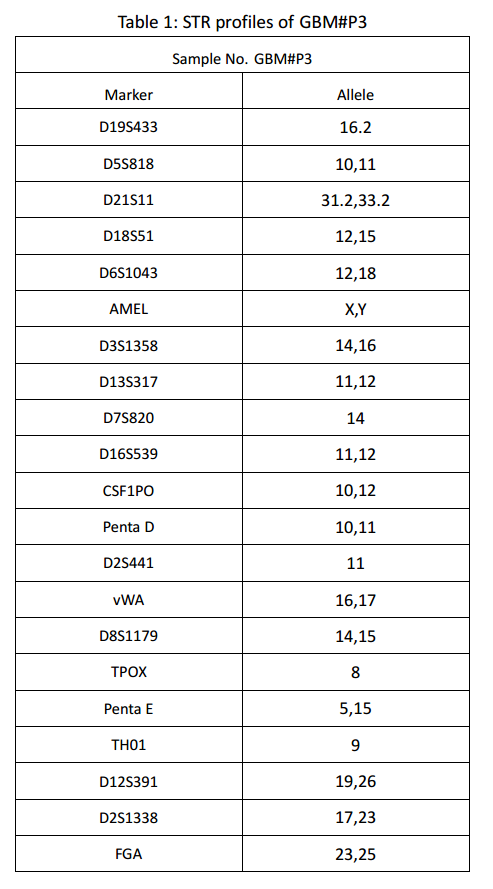


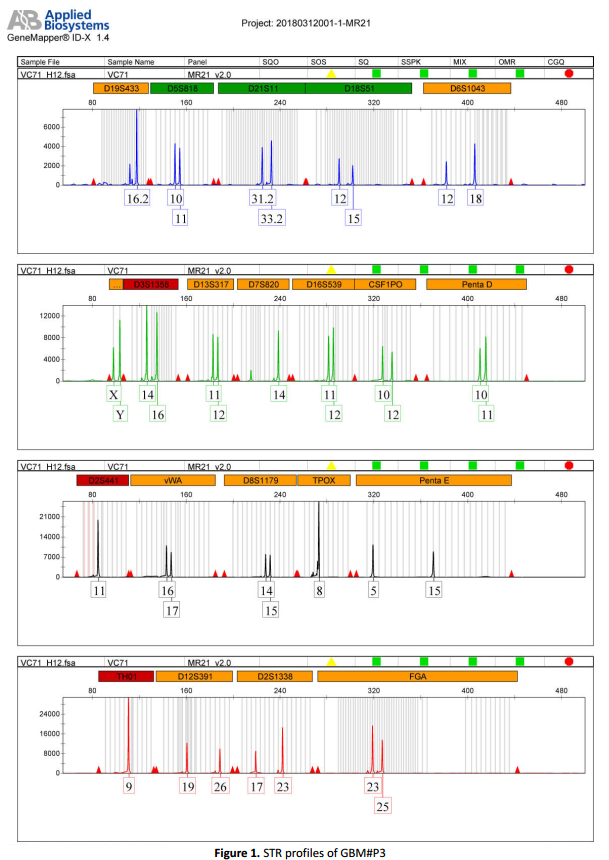


### Table S3. siRNA sequences used in this study

| Gene | Sequence (5’-3’) |
| --- | --- |
| si-PHLPP2#1 | GACCUCUUCAGAUCGUUUATT |
| si-PHLPP2#2 | CUUGGGUUGUUUCCUAUAUTT |

### Table S4. miRNA sequences used in this study

| miRNA | Sequence (5’-3’) |
| --- | --- |
| hsa-miR-25-3p mimics | CAUUGCACUUGUCUCGGUCUGA |
| hsa-miR-25-3p inhibitor | UCAGACCGAGACAAGUGCAAUG |

### Table S5. qRT-PCR primers used in this study

| Gene | Forward (5'-3') | Reverse (5'-3') |
| --- | --- | --- |
| Hsa-miR-25-3p | CCGCGCATTGCACTTGTC | TATGGTTGTTCACGACTCCTTCAC |
| Mus-miR-25-3p | CCGCGCATTGCACTTGTC | TATGGTTGTTCACGACTCCTTCAC |
| Hsa-ARG-1 | TGACGGACTGGACCCATCTT | GGCTTGTGATTACCCTCCCG |
| Hsa-CD163 | GGGCTAATTCCAGTGCAGGT | GCTGACTCATTCCCACGACA |
| Hsa-TNF-α | GAGGCCAAGCCCTGGTATG | CGGGCCGATTGATCTCAGC |
| Hsa-PHLPP2 | TGCTCCACAAAAGGAGGGG | CAATTTCTGCTCCCATTGCGT |
| Mus-Arg-1 | TGTCCCTAATGACAGCTCCTT | GCATCCACCCAAATGACACAT |
| Mus-Cd206 | ACGAGCAGGTGCAGTTTACA | ACATCCCATAAGCCACCTGC |
| Mus-Tnf-α | CGGGCAGGTCTACTTTGGAG | ACCCTGAGCCATAATCCCCT |
| Mus-Phlpp2 | CACGACCACTGCCACTACAA | CAATCTGGAGAGGCCGTTCA |

### Table S6. Antibodies used in this study

| Antibodies | Manufacturer | Cat No. |
| --- | --- | --- |
| CD9 | Cell Signaling Technology | 13403S |
| CD63 | Abcam | ab216130 |
| CD81 | Cell Signaling Technology | 52892S |
| TSG101 | Abcam | ab125011 |
| Calnexin | Cell Signaling Technology | 2679S |
| ARG-1 | Proteintech | 16001-1-AP |
| GAPDH | Proteintech | 10494-1-AP |
| Ki67 | Servicebio | GB111499 |
| CD163 | Abcam | ab182422 |
| P-AKT (Ser473) | Cell Signaling Technology | 4060S |
| AKT | Cell Signaling Technology | 4691S |
| mTOR | Proteintech | 66888-1-Ig |
| P-mTOR | Cell Signaling Technology | 5536S |
| PHLPP2 | Abcam | ab71973 |
| APC Anti-CD11b | Abcam | ab239292 |
| PE Anti-Human CD163 | Abcam | ab95613 |
| CoraLite® Plus 488-conjugated CD206 | Proteintech | CL488-18704 |
